# Supplementary material for: Study protocol for development and validation of a single tool to assess risks of stroke, diabetes mellitus, myocardial infarction and dementia: DemNCD-Risk
Source: BMJ Open. 2023 Sep 22;13(9):e076860. doi: 10.1136/bmjopen-2023-076860 (PMC10533692; doi:10.1136/bmjopen-2023-076860)
Supplement: Author's manuscript [file bmjopen-2023-076860.draft_revisions.pdf]

BMJ Open

BMJ Open is committed to open peer review. As part of this commitment we make the peer review history of every article we publish publicly available.

When an article is published we post the peer reviewers' comments and the authors' responses online. We also post the versions of the paper that were used during peer review. These are the versions that the peer review comments apply to.

The versions of the paper that follow are the versions that were submitted during the peer review process. They are not the versions of record or the final published versions. They should not be cited or distributed as the published version of this manuscript.

BMJ Open is an open access journal and the full, final, typeset and author-corrected version of record of the manuscript is available on our site with no access controls, subscription charges or pay-per-view fees (http://bmjopen.bmj.com).

If you have any questions on BMJ Open's open peer review process please email info.bmjopen@bmj.com

BMJ Open

Study protocol for development and validation of a single tool to assess risks of stroke, diabetes mellitus, myocardial infarction, and dementia: DemNCD-Risk

Journal:	*BMJ Open*
Manuscript ID	bmjopen-2023-076860
Article Type:	Protocol
Date Submitted by the Author:	19-Jun-2023
Complete List of Authors:	Kootar, Scherazad; Neuroscience Research Australia; University of New South Wales, School of Psychology<br>Huque, Md Hamidul; University of New South Wales, School of Psychology; Neuroscience Research Australia<br>Kiely, Kim; University of New South Wales, School of Psychology; Neuroscience Research Australia<br>Anderson, Craig; George Institute for Global Health, The George Institute for Global Health<br>Jorm, Louisa; University of New South Wales, Centre for Big Data Research in Health<br>Kivipelto, Miia; Karolinska Institutet, Division of Geriatric Epidemiology<br>Lautenschlager, Nicola; University of Melbourne Academic Unit for Psychiatry of Old Age, Academic Unit of Psychiatry of Old Age<br>Matthews, Fiona; Newcastle University, Population Health Sciences Institute<br>Shaw, Jonathan; Baker Heart and Diabetes Institute<br>Whitmer, Rachel; University of California Davis<br>Peters, Ruth; University of New South Wales,<br>Anstey, Kaarin; University of New South Wales, School of Psychology; Neuroscience Research Australia
Keywords:	Risk Factors, Primary Prevention, Dementia, Stroke < NEUROLOGY, DIABETES & ENDOCRINOLOGY, Myocardial infarction < CARDIOLOGY

SCHOLARONE™
Manuscripts

Study protocol for development and validation of a single tool to assess risks of stroke, diabetes mellitus, myocardial infarction, and dementia: DemNCD-Risk

Scherazad Kootar[1,2,3], Md Hamidul Huque[1,2,3], Kim Kiely[1,2,3], Craig S. Anderson[5], Louisa Jorm[6], Miia Kivipelto[7,8,9,10], Nicola T. Lautenschlager[11,12], Fiona E Matthews[13], Jonathan E. Shaw[14], Rachel A. Whitmer[15], Ruth Peters[2,3,4,5], *Kaarin J. Anstey[1,2,3]

1. Neuroscience Research Australia, Sydney, Australia
2. School of Psychology, University of New South Wales, Sydney, Australia
3. UNSW Ageing Futures Institute, University of New South Wales, Sydney, Australia
4. School of Public Health, Imperial College, London, Exhibition Road, South Kensington, London SW 2AZ UK
5. The George Institute for Global Health, University of New South Wales, Sydney, Australia
6. Centre for Big Data Research in Health, University of New South Wales, Sydney, Australia
7. The Ageing Epidemiology Research Unit, School of Public Health, Imperial College London, London, United Kingdom
8. Division of Clinical Geriatrics, Department of Neurobiology, Center for Alzheimer's Research, Care Sciences and Society, Karolinska Institute, Stockholm, Sweden
9. Theme Inflammation and Aging, Karolinska University Hospital, Stockholm, Sweden
10. Institute of Public Health and Clinical Nutrition, University of Eastern Finland, Kuopio, Finland
11. Academic Unit for Psychiatry of Old Age, Department of Psychiatry, The University of Melbourne, Parkville, Australia
12. North Western Mental Health, Royal Melbourne Hospital, Parkville, Australia
13. Population Health Sciences Institute, Newcastle University, UK
14. Baker Heart and Diabetes Institute, Melbourne, Australia
15. Department of Public Health Sciences and Neurology, University of California, Davis CA 95616, USA

***Corresponding author**:

Scientia Professor Kaarin J. Anstey
School of Psychology,
University of New South Wales,
Kensington NSW 2052, Australia.
Email: k.anstey@unsw.edu.au
Telephone no: +61 9399 1061

Word count: 3,423

For peer review only - http://bmjopen.bmj.com/site/about/guidelines.xhtml

ABSTRACT

Introduction

Current efforts to reduce dementia focus on prevention and risk reduction by targeting modifiable risk factors. As dementia and cardiometabolic non-communicable diseases (NCDs) share risk factors, a single risk-estimating tool for dementia and multiple NCDs could be cost-effective and facilitate concurrent assessments as compared to a conventional single approach. The aim of this study is to develop and validate a new risk tool that estimates an individual's risk of developing dementia and other NCDs including diabetes mellitus, stroke, and myocardial infarction. Once validated, it could be used by the public and General Practitioners.

Methods and Analysis

Ten high-quality cohort studies from multiple countries were identified that met eligibility criteria, including large representative samples, long-term follow-up, data on clinical diagnoses of dementia and NCDs, recognised modifiable risk factors for the four NCDs, and mortality data. Pooled harmonized data from the cohorts will be used, with 65% randomly allocated for development of the predictive model and 35% for testing. Predictors include social-demographic characteristics, general health risk factors, and lifestyle/behavioural risk factors. A sub-distribution hazard model will assess the risk factors' contribution to the outcome, adjusting for competing mortality risks. Point-based scoring algorithms will be built using predictor weights, internally validated, and the discriminative ability and calibration of the model will be assessed for the outcomes. Sensitivity analyses will include recalculating risk scores using logistic regression.

Ethics and dissemination

Ethics approval is provided by the University of New South Wales Human Research Ethics Committee (UNSW HREC; protocol numbers HC200515, HC3413). All data are de-identified and securely stored on servers at Neuroscience Research Australia. Study findings will be presented at conferences and published in peer-reviewed journals. The tool will be accessible as a public health resource. Knowledge translation and implementation work will explore strategies to apply the tool in clinical practice.

Keywords: risk prediction tool, dementia, stroke, diabetes mellitus, myocardial infarction

For peer review only

For peer review only - http://bmjopen.bmj.com/site/about/guidelines.xhtml

Strengths and limitations of this study

- This international and multi-cohort collaboration draws on data from multiple countries including Australia, Netherlands, Singapore, UK, and USA.
- To our knowledge this will be one of the first validated risk-prediction tools for multiple NCDs, including dementia, that accounts for competing risks and draws on datasets from diverse geographic regions.
- External validation will be required to assess generalisability to under-represented communities, low-resource settings, and other populations not included in the contributing studies.
- We rely on post-hoc harmonisation of risk factors, and not all risk factor measures are directly comparable across studies.

For peer review only

For peer review only - http://bmjopen.bmj.com/site/about/guidelines.xhtml

INTRODUCTION

Non communicable diseases (NCDs) are a global health issue leading to around 41 million deaths each year. Amongst these, cardiovascular diseases account for most deaths (17.9 million), followed by cancer (9.3 million), respiratory illness (4.1 million) and diabetes (1.5 million) (1). According to the Centers for Disease Control and Prevention, USA, dementia ranked seventh for mortality in 2020, alongside the above diseases partially due to global population ageing (2). Dementia is the largest contributor to the non-fatal burden of disease amongst older adults in Australia (3). The Global Burden of Diseases, Injuries, and Risk Factors (GBD) Study 2019 (4) reports the estimated global number of people with dementia increasing from 57 million in 2019 to 153 million in 2050. This increase in the number of people with dementia will negatively impact on individuals, families, health care systems, and society. Given that there is no cure or effective treatment to stop the progression of dementia, the field has shifted focus towards prevention and risk reduction to reduce the burden on health and social care systems. Evidence on modifiable risk factors for dementia has been comprehensively synthesized into recent umbrella reviews (5, 6), a Lancet Commission (7, 8) and WHO Guidelines (9). These reviews show that many NCDs are also risk factors for dementia, including diabetes mellitus (DM), stroke, and other cardiovascular diseases (CVD) (5, 6). In addition, these four NCDs also share common social and lifestyle related risk factors (10). Given the overlap in risk between dementia and other NCDs, the development of common risk reduction strategies may be more cost-effective and efficient, than conventionally approaching each disease individually.

For peer review only - http://bmjopen.bmj.com/site/about/guidelines.xhtml

Risk assessments for NCDs are widely used in clinical practice, research, and policymaking (11-14). The Framingham Risk Score, a well-known example of a health risk assessment for cardiovascular disease risk, was adapted to calculate the absolute CVD risk and is used in the clinical practice guidelines and recommendations of many countries to guide treatment for the primary prevention of CVD (15). Risk assessments primarily identify individuals at high risk of developing the disease and determine preventive measures for reducing risk. They are also used to evaluate risk reduction interventions (15, 16), and population modelling of risk levels (17, 18). Currently there are numerous risk tools available for dementia (19), DM (20), heart disease (21) and stroke (22). Examples of scoring systems used to predict dementia include the Cardiovascular Risk Factors, Aging, and Incidence of Dementia (CAIDE), the ANU-Alzheimer's Disease Risk Index (ANU-ADRI), and the Cognitive Health and Dementia Risk Reduction (CogDrisk) (23-26). Similarly, for the cardiometabolic field there are Framingham risk scores for cardiovascular disease (10-year and 30-year risk) (27, 28), the Australian type 2 Diabetes Risk Assessment tool (AUSDRISK) (29), the FINDRISC Diabetes risk tool (30), the Cambridge Diabetes Risk Score (31), the Framingham diabetes risk score to assess risk of diabetes mellitus (29, 32) and the Framingham stroke risk score (22). Variables such as lack of physical activity, unhealthy diet, smoking, hypertension, and obesity are relevant to dementia and overlap with risk factors for diabetes mellitus and cardiovascular diseases. To our knowledge there is no single risk prediction tool for NCDs that includes dementia. Building such a tool that accounts for substantial overlap in risk factors and interactions between NCDs may be cost-effective and save clinical assessment time as compared to assessing each disease individually.

For peer review only - http://bmjopen.bmj.com/site/about/guidelines.xhtml

To address the need for a population-based prediction tool for incidence of multiple NCDs including dementia, we propose the development and validation of the DemNCD-Risk tool. This risk tool will provide a combined estimate for an individual's risk of developing dementia, type 2 diabetes mellitus, stroke and myocardial infarction. We anticipate that a multi-domain risk tool that accounts for shared and competing risk will be more likely to be used in primary care as it would reduce assessment time. This study protocol outlines the analytical approach used to develop and validate our risk tool.

METHODS AND ANALYSIS

Study design

Prior to commencing the study, we determined the minimum sample size required and types of cohorts that would be suitable. Inclusion criteria were: population-based cohort studies of mid-life and/or older age participants, with at least 5 years follow-up (exception being CFAS-II with follow up time of 2 years), having clinical diagnosis of dementia and other NCDs and the availability of the majority of risk factors used in current prediction tools. We included CFAS-II due to its similarity to CFAS-I in terms of being based in England and having a comparable population and characteristics. Moreover, the availability of accessible data of CFAS-II along with CFAS-I supported our rationale for including this study in our analysis.

We identified potential datasets through searches of consortia websites, databases, word of mouth, and consultation with experts. We evaluated cohorts in terms of the outcome measures including studies with the availability of clinical diagnosis of dementia and other NCDs, risk factors and measures (which will also be referred to as predictors in the

For peer review only - http://bmjopen.bmj.com/site/about/guidelines.xhtml

methodology), length of follow-up time, sample size requirements, and the availability of data from the study custodians. We shortlisted ten cohort studies that met our criteria: the Cardiovascular Health Study (CHS) (33), the Framingham Heart Study (FHS) (34-36), the MRC Cognitive Function and Ageing Studies (CFAS-I (37, 38) and CFAS-II (39)), the Sydney Memory and Aging Study (MAS) (40), the Maastricht Aging Study (MAAS) (41, 42), the Health and Retirement Study- Aging, Demographics and Memory Study (HRS ADAMS) (43-45), the Memory and Aging Project (MAP) (46), the Atherosclerosis Risk in Communities (ARIC) (47, 48) and the Singapore Longitudinal Ageing Studies-I (SLAS I) (49, 50). Key characteristics for each study are available in Table 1. Ethics approval was obtained from the UNSW Human Research Ethics Committee (approval number HC00515) and approval from the data custodians were obtained to access the datasets.

We report the study protocol following the Transparent Reporting of a multivariable prediction model for Individual Prognosis or Diagnosis (TRIPOD) statement for prediction studies.

Participants

Individual data will be pooled from the ten cohort studies (age 44 to 110 years). Participants from these cohorts who have dementia at baseline will be excluded.

Outcomes

The primary objective of the current study is to develop a validated risk assessment tool to predict four different NCDs including dementia, myocardial infarction (MI), stroke and DM. We chose to predict for MI instead of coronary heart disease (CHD) as only CHS and FHS had a composite variable for CHD which was adjudicated by a subcommittee while MI was reported in all studies. These NCDs were chosen as they are highly

For peer review only

For peer review only - http://bmjopen.bmj.com/site/about/guidelines.xhtml

prevalent, related, and they share common modifiable risk factors that are often assessed and managed in primary care. Clinical diagnosis of each of these outcomes will be used (where available), however self-reports of diagnosis may also be used for stroke, diabetes, and MI. Detailed descriptions of each of these outcomes are presented below and described in Table 3:

Dementia: All included studies diagnosed dementia through the Diagnostic and Statistical Manual of Mental Disorders (DSM-III-R, IV criteria) or International Classification of Diseases (ICD) codes. We chose to study all- type dementia instead of specific types for two reasons: one, as there are no tools available to understand the different dementia pathologies to predict dementia types; and two, as we intend to build a tool, which is simple and facilitates public participation. Algorithms were used for diagnosis of dementia in ARIC (48) and CFAS (51).

Stroke: For the ARIC study, a diagnostic algorithm created variable that classifies stroke events based on data from reports will be used to define stroke. For the CHS, special cerebrovascular subcommittees defined the criteria for stroke and TIA (52) while in the FHS, stroke cases were reviewed by neurologists. Where clinical diagnosis is not available, for example CFAS I and II, Sydney MAS, SLAS-I and MAAS, and HRS ADAMS self-reported stroke will be used.

Diabetes mellitus: Similar to stroke, clinical diagnosis of DM (including type of diabetes where information is available), derived variables or lab results will be preferred over self-reported DM. Derived variables for diabetes based on WHO guidelines or American Diabetes Association guidelines are available in the CHS while self-reported type 2 diabetes will be considered for other studies.

For peer review only - http://bmjopen.bmj.com/site/about/guidelines.xhtml

MI: All the studies report self-reported heart attacks.

Death: Date/year of death or age at death are available in all studies.

Predictors

We identified model predictors in three stages. Firstly, we identify predictors from the latest seminal systematic reviews for the four outcomes (5, 6, 10, 53-57), the Lancet Commission on dementia (7, 8), and the WHO Guidelines for evidence on risk factors and risk reduction of cognitive decline and dementia (9). Secondly, predictors from commonly used risk tools were collated into a long list. For this, we referred to the ANU-ADRI, LIBRA, and CAIDE for dementia predictors, the Framingham hard coronary heart disease risk tool (10-year risk for myocardial infarction or coronary death), Framingham risk score for cardiovascular disease (10-year and 30- year), and CVD check for myocardial infarction predictors, the AUSDRISK and Framingham diabetes risk score for diabetes predictors, and the Framingham stroke risk score for stroke predictors (refer table 2). Lastly, the long list of potential predictors was then reviewed and ranked by seven subject matter experts who had previous experience with population-based prediction models (24, 29). Ethics approval was obtained from UNSW Human Research Ethics Committee (protocol number HC3413). We received five rankings for dementia predictors, three each for predictors of stroke, diabetes and myocardial infarction. In total, we identified 18 predictors for stroke, 15 for diabetes, 18 for coronary heart disease and 23 for dementia. Predictors were extracted from the baseline examination or from the first wave in which they were measured.

Candidate predictors considered for inclusion in the predictive model (definitions are provided in Appendix Table 4):

For peer review only - http://bmjopen.bmj.com/site/about/guidelines.xhtml

- *Demographic factors*: age, sex, education level, ethnicity/country of birth, socioeconomic status, and family history.
- *Lifestyle risk/protective factors*: smoking, harmful alcohol consumption, fish intake/healthy diet, insufficient physical activity, social isolation, lack of cognitive engagement and sleep disturbance.
- *Medical risk factors*: mid-life obesity, hypertension, hypercholesterolemia, triglycerides, low and high density lipoproteins, late-life atrial fibrillation, chronic kidney disease, high blood glucose, urinary albumin and protein, left ventricular hypertrophy, depression, traumatic brain injury, high homocysteine, stress, orthostatic hypotension, and hearing loss.

Table 4 reports the risk factors and their definitions.

Sample size

We assumed that the proposed risk algorithms that generate risk scores for dementia and other outcomes will have sensitivity between 70% and 90% across different cohorts as the prevalence of disease will vary across cohorts. Assuming global prevalence of dementia at age 60 and older is 5%, we estimated that 9 studies with sample sizes between 1,041 and 2,165 will be needed to obtain a sensitivity of 70% and 90% respectively with 10% precision and 5% level of significance with Bonferroni correction. We focused on dementia for sample size calculations, as among all the outcomes considered for this study, dementia has the lowest prevalence. Therefore, the minimum sample size for dementia will be sufficient to provide required statistical power for other outcomes.

For peer review only - http://bmjopen.bmj.com/site/about/guidelines.xhtml

Statistical analysis methods

Analysis plan

We will pool harmonized individual data from the ten studies to develop a risk tool that provides long term risk estimates for diabetes, stroke, MI, and dementia. Machine learning methods will be used to develop and validate the risk tools. Specifically, we will use 65% of the pooled data in training the model, and the remaining 35% of the data will serve as test data for internal validation in order to ensure good coverage of the outcomes in the validation sample. We plan to validate the tool on additional datasets in future work. As our objective is to build a tool that predicts long term health outcomes, we will use competing risk survival models (Fine and Gray sub-distribution hazard model (58)). When predicting outcomes over long periods of time competing risks should account for risk of death from non-disease related causes as this risk increases over time and decedents are no longer at risk of a disease incidence. As the competing risk of mortality is more common in high-risk populations with a long duration of follow-up, this effect may be more pronounced in older cohorts than in younger or healthy cohorts. Moreover, this model allows one to estimate the effect of covariates on the cumulative incidence for the event of interest. Predictor weights from this model for risk factor exposure and their interactions will be combined to build point-based scoring algorithms that provide individualized risk prediction (59). The individual risk scores will further be validated internally using the area under the curve (AUC) (60). Model calibration will also be carried out. We will estimate sensitivity and specificity of each risk algorithm based on a suitable cut-off on the ROC curve.

For peer review only - http://bmjopen.bmj.com/site/about/guidelines.xhtml

Data harmonisation and coding of predictors

Each study wave will be screened for the availability of risk factors and outcomes using the data dictionaries. Predictors and outcomes listed above will be checked for availability of the measure in each wave of the different datasets. Data harmonisation will be carried out as described previously (61) using the 'by fiat' method (62). This method is developed using a common scoring system across studies to standardize the measure of the variables. In certain cases, for example cognitive engagement, we will capture the total cognitive activity per study as compared to harmonising the variable across the studies as different items of cognitive activity are measured in each study. Detailed definitions and measurements of the predictors are presented in Table 4.

We will use national / international guidelines where possible, for example; 150 minutes of moderate and vigorous physical activity per week which is equivalent to >500 MET minutes per week of physical activity (63), 5 or more servings of vegetables or 3 -5 servings of fruit and vegetables and more than 2 servings of fish per week will be used to represent a low risk diet (as measured by the AHA's Life's Simple 7 guidelines (64), alcohol consumption of not more than 10 standard drinks (1 standard drink = 10 grams of pure alcohol) per week as recommended by the Australian guidelines (65). For biological markers, we will use cut-offs informed by national and international clinical practice guidelines, and recommendations from national healthcare providers for example the Royal Australia College of General Practitioners (13, 66, 67). Cut-offs to be used: high-density lipoprotein (<1 mmol/L for men and <1.3 mmol/L for women), low density lipoprotein (>4.1 mmol/L), high cholesterol (>6.18mmol/L) and triglycerides (>2.3mmol/L). We will choose "high" instead of "borderline high" as the most robust category for the

For peer review only - http://bmjopen.bmj.com/site/about/guidelines.xhtml

prediction of disease outcomes. Self-report stress measures will be used, clinical thresholds for high homocysteine, urinary albumin and protein if reported in articles from the cohort studies to be included.

Missing data

Missing data are inevitable in studies with long follow-up and may lead to bias. Variables with missing data will be imputed through multiple imputation across all studies (68). Specifically, a multivariate normal imputation (MVNI) or imputation by fully conditional specification, where appropriate, will be employed to impute missing data. Compatibility/congeniality between imputation model and analysis model will be ensured. The number of imputations will be determined according to the current guidelines (69).

Model specification

Following multiple imputation, competing risk regression with available covariates (candidate covariates that are available in the dataset) and death as a competing event will be modelled for each outcome, stratified by sex. Robust sandwich estimation of the variance will be obtained. All the cohorts under consideration are likely to be heterogeneous in terms of sample sizes and underlying population characteristics i.e., outcome and covariate distribution. Moreover, running competing risk analysis with multiple imputed datasets on large number of data may cause the slow convergence of the program. Therefore, we will run the competing risk regression model in each of the datasets separately. As we plan to evaluate the competing risk model in individual datasets, we will not be able to create 10-year risk prediction estimates.

For peer review only - http://bmjopen.bmj.com/site/about/guidelines.xhtml

Model estimation

Estimates of the regression models for multiple imputed models will be combined using Rubin's rule in each cohort. The resulting regression estimates will be combined across cohorts to get final estimates using meta regression. The final regression coefficients estimates will then be used to calculate the points-based risk score for males and females separately, which we will then test with the testing datasets for prediction of each of the outcomes (60). All the analyses will be carried out using Stata Version 16.0.

Assessment of Predictive performance

Area under the curve (AUC), sensitivity and specificity of the risk scores on the test dataset will be obtained to assess the discriminatory power. We will also assess the calibration of the risk algorithms using calibration plots and Hosmer-Lemeshow statistics (70).

Model presentation

The final regression coefficients (95% CI) for dementia, stroke, diabetes and MI, along with the points corresponding to the final algorithm will be made available.

Analysis beyond initial model development

In addition to the complex survival model, we will also calculate risk scores from the cohorts under consideration using logistic regression to test for the effect of follow-up time on risk scores. Specifically, we will be modelling binary outcomes with all the predictors ignoring follow-up time for the initial model. The risk score will then be calculated using the regression coefficients from the logistic model using a similar methodology.

For peer review only - http://bmjopen.bmj.com/site/about/guidelines.xhtml

For peer review only

In addition to the above model with all the risk factors, we are also planning to build a model based on the best subset of predictors. Specifically, for each of outcome, we are planning to perform backward elimination ($p<0.1$). The risk scores for each outcome will then be calculated using the regression coefficients obtained from the most parsimonious model. The AUC (95% CI) of the most parsimonious model will then be compared with the AUC obtained from the full model.

Translation of the analyses into a risk assessment for implementation

To generate information on risk of dementia, DM, stroke and MI using a single assessment, we will create a single set of questions that encompass all the risk factors so that the patient experience is a single assessment. Algorithms will be applied to this tool to generate advice and risk estimates for multiple outcomes. Ideally the analyses of the cohort studies will provide a risk algorithm for each outcome. However, this may be supplemented by existing risk algorithms if the results of the proposed analyses are not sufficient. Where possible, risk factors will be assessed as measured in the original cohort studies. Where the quality of measures is mixed or of suboptimal quality, we will identify validated measures. For example, with respect to the measurement of insomnia, some studies use clinical sleep scales and others include a study-specific self-report item. We will use a standardized and validated instrument for diagnosis of insomnia in the assessment tool. We anticipate that two assessment tools will be created: one for the clinicians and another for researchers and the public.

For peer review only - http://bmjopen.bmj.com/site/about/guidelines.xhtml

LIMITATIONS

Our study has some limitations. Although using multiple cohorts to develop the tool is an improvement compared with a single cohort, our cohorts are drawn mainly from high-income countries and with most of them being mainly Caucasian due to the availability of these datasets with the required information. This will limit the generalizability of the tool. We are unable to take account of the differences in treatment and care that will have influenced participants in different cohorts.

However, as the tool will be developed and validated from data pooled from America, Asia, Australia and Europe, we have some diversity within our sample. As the tool will be validated on the same sample as the derivation cohort, external validation of the tool will be desirable after study completion. We are unable to account for cause of death in the analysis as most of the studies lack this information.

ETHICS AND MODEL DISSEMNINATION

Ethics approval is provided by the University of New South Wales Human Research Ethics Committee (UNSW HREC; protocol numbers HC200515, HC3413). All data are de-identified and stored on a secure server at Neuroscience Research Australia. Study findings will be presented at relevant national and international conferences and published in peer review journals. The tool will be made available as a public health resource. Knowledge translation and implementation work will explore strategies to apply the tool in clinical practice.

For peer review only - http://bmjopen.bmj.com/site/about/guidelines.xhtml

CONCLUSION

We aim to develop and validate a single tool to predict multiple NCDs including dementia, diabetes, myocardial infarction, and stroke. This tool may be more feasible than individual risk assessment tools, facilitate preventive health assessments, and more importantly will save clinical assessment time.

For peer review only

For peer review only - http://bmjopen.bmj.com/site/about/guidelines.xhtml

Authors' contributions

KJA is the lead investigator of this study and oversaw the project development. KJA and RP developed the original project idea. KJA, RP, KK, HH and SK developed the analysis plan, study design including identifying various cohort studies. FM, LJ, RW and JS provided additional inputs to the analysis plan. SK and KJA applied to various studies and got approval. SK reviewed the literature, conducted the survey on subject matter experts for selecting predictors and prepared the first draft of the manuscript. SK, KJA, KK, RP and HH planned the harmonisation of outcomes and predictors. HH drafted the method section. All authors critically reviewed and contributed to the protocol and approved the final draft.

Funding

This research was funded by a NeuRA Grant to KJA, the NHMRC Dementia Collaborative Research Centre, and NHMRC GNT1171279. KJA is funded by the Australian Research Council Fellowship FL190100011, SK and HH are part funded by NHMRC GNT1171279. KK is part funded by ARC CE170100005. JES is supported by NHMRC Investigator Grant APP1173952. CSA holds research grants and a Senior Investigator Fellowship from the NHMRC.

Competing interests

JES has received honoraria for scientific advisory, lectures, and clinical research from Pfizer; Roche; Zuellig Pharma; Astra Zeneca; Sanofi; Novo Nordisk; MSD; Eli Lilly; Abbott; Mylan; Boehringer Ingelheim. CSA has received grants from Takeda, Penumbra and Credit pharma awarded to his institution outside of this work.

For peer review only - http://bmjopen.bmj.com/site/about/guidelines.xhtml

Table 1: Characteristics of contributing studies

Study	Baseline year	Age range at baseline	Number of individuals at baseline	Males (n) and Females (n)	Number of waves and year	Follow up time	Country
Maastricht Aging Study (MAAS)	1992	24 - 81 years	1,823	Women= 910, Men=913	Baseline (1992-1996), Wave 1 (1996-1999), Wave 2 (1999-2002), Wave 3 (2002-2005), Wave 4 (2005-2008) Wave 5 (2018-2020)	25 years	The Netherlands
The Framingham Heart Study (FHS)	1948	30 -62 years	5,209	Women =2,873, Men =2,336	Wave 1 (1948-1953) to Wave 32 (2012-2014)	61 years	USA
Atherosclerosis Risk in Communities (ARIC)	1985	44-66 years	15,028	Women =8,198, Men =6,830	Wave 1 (1987-1989), Wave 2 (1990-1992), Wave 3 (1993-1995), Wave 4 (1996-1998), Wave 5 (2011-2013), Wave 6 (2015-2016), Wave 7 (2017-2018), Wave 8 (2019-2020)	31 years	USA
Health and Retirement Study - Aging, Demographics and Memory Study (HRS ADAMS)	1992	70 years and above	856	Women =501, Men=355	Wave 1 (2002-2005), Wave 2 (2006-2008), Wave 3 (2008-2009)	8 years	USA
The Singapore Longitudinal Ageing Study (SLAS I)	2003	55 years and above	2,567	Women =1,666, Men= 901	Wave 1 (2003-2005) and Wave 2 (2007-2009)	6 years	Singapore

For peer review only

The Cardiovascular Health Study (CHS)	1991-1994	65-100 years	5,888	Women =3,393, Men =2,495	Wave 1 (1991-1994) Final follow-up (1999-2000)	8 years	USA
Cognitive Function and Ageing Study (CFAS-I)	1989-1991	65 -105 years and above	13,004	Women =7,847, Men = 5,157	Wave 1 (1991-1994), Wave 2 (1991-1995), Wave 3 (1992-1996), Wave 4 (1994-1998), Wave 5 (1997-2001)	10 years	UK
Cognitive Function and Ageing Study (CFAS-II)	2008	65 -100 years and above	7,762	Women =4,228, Men =3,534	Wave 1 (2008-2011) Wave 2 (2011-2013)	2 years	UK
Sydney Memory and Aging Study (MAS)	2005	70-90 years	1,037	Women = 572, Men =465	Wave 1 (2005-2007), Wave 2 (2007-2009), Wave 3 (2009-2011), Wave 4 (2011-2013), Wave 5 (2013-2016), Wave 6 (2016-2018), Wave 7 (2018 onwards)	12 years	Australia
Rush Memory and Aging Project (MAP)	1997	55 years and above	2,192	Women =1,610, Men =582	Wave 1 (1998) to Wave 22 (2019 and onwards)	22 years	USA

For peer review only - http://bmjopen.bmj.com/site/about/guidelines.xhtml

Table 2: List of predictors included in current risk tools for dementia, diabetes, myocardial infarction and stroke

Risk factor category	Dementia risk tools (CAIDE, ANU ADRI, LIBRA)	Cardiovascular risk tools (Framingham Risk Tools- Hard Coronary Heart Disease, 10- /30-year risk of CVD, CVD Check)	Diabetes risk tools (AUSDRISK, Framingham risk score for Diabetes)	Stroke risk tool (Framingham stroke risk score)
Demographic variables				
Age and Sex	Y	Y	Y	Y
Education	Y	-	-	-
Ethnicity	-	Y	Y	-
Parental /family history of disease	-	Y	Y	-
Medical risk factors				
Hypertension/ Systolic BP	Y	Y	Y	Y
Use of antihypertensives	-	Y	Y	Y
High total cholesterol	Y	Y	-	-
HDL cholesterol	-	Y	Y	-
Obesity/ Waist circumference	Y	Y	Y	-
Diabetes	Y	Y	-	Y
Depression	Y	-	-	-
Traumatic Brain Injury	Y	-	-	-
High blood glucose levels	-	-	Y	-
Triglycerides	-	-	Y	-
Chronic kidney disease/renal dysfunction	Y	Y	-	-
Urine for microalbumin and protein	-	Y	-	-
Atrial fibrillation	-	Y	-	Y
Prior cardiovascular disease	Y	-	-	Y

For peer review only - http://bmjopen.bmj.com/site/about/guidelines.xhtml

Left ventricular hypertrophy	-	-	-	Y
Lifestyle and behavioural risk factors				
Smoking	Y	Y	Y	Y
Physical inactivity	Y	Y	Y	-
Diet (Fish intake, Consumption of vegetables and fruits)	Y	Y	Y	-
Alcohol consumption	Y	Y	-	-
Social engagement	Y	-	-	-
Cognitive engagement	Y	-	-	-
Environmental exposure				
Pesticide exposure	Y	-	-	-

For peer review only

Table 3: Considerations for defining the outcomes

No	Outcome	Considerations
1	Dementia	Clinical diagnosis of dementia
2	Stroke	Clinical diagnosis of stroke, if not available then self-reported stroke
3	Diabetes	Clinical diagnosis of diabetes or derived variable or lab results, if not available then use self-reported Diabetes.
4	MI	Clinical diagnosis of myocardial infarction, if not available use self-reported questions

For peer review only

For peer review only - http://bmjopen.bmj.com/site/about/guidelines.xhtml

Table 4: Considerations for defining the candidate predictors

Predictors	Considerations
Demographic factors	
Education	Less than secondary vs Secondary and vocational training vs Tertiary
Ethnicity and country of birth	Questions related to race/ethnicity and country of birth
Socioeconomic status	Questions on occupation, income and accommodation
Family history	Family History of stroke, diabetes, dementia, CHD
Lifestyle risk factors	
Smoking status	Current vs Former vs Never
Alcohol consumption	Abstinence vs drinker<br>Average number of drinks per drinking session<br>Heavy drinker vs non heavy drinker
Fish intake / Healthy diet	Fish intake- more than twice per week<br>Fruit and vegetable intake (at least 5 servings of vegetables or 3 servings of vegetables and 2 servings of fruits)
Physical inactivity	Total time per week of physical activity- more than 500 MET per week -physically active vs inactive<br>Sedentary- yes versus no
Social isolation	Loneliness versus not lonely
Cognitive engagement	Decided to use the Total cognitive activity as reported by each study and then to divided into tertiles/quartiles- depending on each study to get a cut-off for cognitive inactivity.
Sleep disturbance	Account for those with at least two symptoms of the three self-reported sleep problems: trouble falling asleep, wake up several times at night, wake up too early
Medical risk factors	
BMI categories	WHO classifications- Underweight (BMI <18.5) vs Normal (BMI 18.5-24.9) vs Overweight (BMI 25-29.9) vs Obese (BMI >30)
Hypertension status	Yes -Blood pressure average systolic >=140mm Hg OR average diastolic >=90 OR history of hypertension OR currently using antihypertensives vs No
High Cholesterol and triglycerides	High cholesterol (self-reported/lifetime history or total cholesterol >6.18 mmol/l)
	High triglycerides (>2.3 mmol/L triglycerides)
	High cholesterol or high triglycerides (self-reported)
Low density lipoproteins	> 4.1 mmol/L of LDL cut-off is high LDL
High density lipoprotein	< 1 mmol/L (for males) and <1.3 mmol/L (for females) of HDL cut-off is low HDL

For peer review only - http://bmjopen.bmj.com/site/about/guidelines.xhtml

Atrial fibrillation	AF measured by ECG or self-reported history of AF
Chronic kidney disease	First preference Clinical diagnosis of kidney disease, if not use self-reported history of kidney disease
High blood glucose	To consider borderline or prediabetes cut off for blood glucose - 100 to 125 mg/dl or 5.6-7.0 mmol/L
Urinary microalbumin and protein	Lab reports of urine microalbumin and protein levels
Left Ventricular Hypertrophy	Yes or no for LVH as defined by study
Depression	Self-reported depression symptoms measured by respective scales of depression used by the respective studies
Traumatic brain injury	Self-reported history of brain injury/head injury/knock out with or without loss of consciousness
High homocysteine	Lab reports of homocysteine levels
Stress	Self-reported stress measures
Orthostatic hypotension	Use self-reported orthostatic hypotension or calculated variable
Hearing loss	To categorise in three groups - no hearing loss, has hearing loss and wears hearing aid, has hearing loss but does not wear a hearing aid.

For peer review only

For peer review only - http://bmjopen.bmj.com/site/about/guidelines.xhtml

References:

1. WHO. Noncommunicable diseases 2021 [Available from: https://www.who.int/news-room/fact-sheets/detail/noncommunicable-diseases.
2. Murphy SL, Kochanek KD, Xu J, et al. Mortality in the United States, 2020. 2021.
3. AIHW. Australian Burden of Disease Study: impact and causes of illness and death in Australia 2018. Cat no BOD 29. 2021.
4. Nichols E, Szoeke CE, Vollset SE, et al. Global, regional, and national burden of Alzheimer's disease and other dementias, 1990–2016: a systematic analysis for the Global Burden of Disease Study 2016. The Lancet Neurology. 2019;18(1):88-106.
5. Anstey KJ, Ee N, Eramudugolla R, et al. A Systematic Review of Meta-Analyses that Evaluate Risk Factors for Dementia to Evaluate the Quantity, Quality, and Global Representativeness of Evidence. Journal of Alzheimer's disease : JAD. 2019;70(s1):S165-s86.
6. Yu J-T, Xu W, Tan C-C, et al. Evidence-based prevention of Alzheimer's disease: systematic review and meta-analysis of 243 observational prospective studies and 153 randomised controlled trials. Journal of Neurology, Neurosurgery & Psychiatry. 2020;91(11):1201-9.
7. Livingston G, Huntley J, Sommerlad A, et al. Dementia prevention, intervention, and care: 2020 report of the Lancet Commission. The Lancet. 2020;396(10248):413-46.
8. Livingston G, Sommerlad A, Orgeta V, et al. Dementia prevention, intervention, and care. The Lancet. 2017;390(10113):2673-734.
9. WHO. Risk Reduction of Cognitive Decline and Dementia: WHO guidelines Geneva: World Health Organisation; 2019.
10. Peters R, Booth A, Rockwood K, et al. Combining modifiable risk factors and risk of dementia: a systematic review and meta-analysis. BMJ Open. 2019;9(1):e022846.
11. Duerden M, O'Flynn N, Qureshi N. Cardiovascular risk assessment and lipid modification: NICE guideline. British Journal of General Practice. 2015;65(636):378-80.
12. Kaptoge S, Pennells L, De Bacquer D, et al. World Health Organization cardiovascular disease risk charts: revised models to estimate risk in 21 global regions. The Lancet Global Health. 2019;7(10):e1332-e45.
13. Litt J. RACGP Guidelines for preventive activities in general practice 9th edition. 2016.
14. Visvanathan K, Hurley P, Bantug E, et al. Use of pharmacologic interventions for breast cancer risk reduction: American Society of Clinical Oncology clinical practice guideline. Journal of Clinical Oncology. 2013;31(23):2942-62.
15. NVDPA. Guidelines for the management of absolute cardiovascular disease risk. 2012.
16. Anstey KJ, Cherbuin N, Kim S, et al. An internet-based intervention augmented with a diet and physical activity consultation to decrease the risk of dementia in at-risk adults in a primary care setting: pragmatic randomized controlled trial. Journal of medical Internet research. 2020;22(9):e19431.
17. Ng R, Sutradhar R, Kornas K, et al. Development and validation of the Chronic Disease Population Risk Tool (CDPoRT) to predict incidence of adult chronic disease. JAMA network open. 2020;3(6):e204669-e.
18. Hajifathalian K, Ueda P, Lu Y, et al. A novel risk score to predict cardiovascular disease risk in national populations (Globorisk): a pooled analysis of prospective cohorts and health examination surveys. The lancet Diabetes & endocrinology. 2015;3(5):339-55.
19. Hou X-H, Feng L, Zhang C, et al. Models for predicting risk of dementia: a systematic review. Journal of Neurology, Neurosurgery & Psychiatry. 2019;90(4):373-9.
20. Noble D, Mathur R, Dent T, et al. Risk models and scores for type 2 diabetes: systematic review. Bmj. 2011;343.

For peer review only - http://bmjopen.bmj.com/site/about/guidelines.xhtml

21. Damen JA, Hooft L, Schuit E, et al. Prediction models for cardiovascular disease risk in the general population: systematic review. bmj. 2016;353.
22. D'Agostino RB, Wolf PA, Belanger AJ, et al. Stroke risk profile: adjustment for antihypertensive medication. The Framingham Study. Stroke. 1994;25(1):40-3.
23. Kivipelto M, Ngandu T, Laatikainen T, et al. Risk score for the prediction of dementia risk in 20 years among middle aged people: a longitudinal, population-based study. The Lancet Neurology. 2006;5(9):735-41.
24. Anstey KJ, Cherbuin N, Herath PM. Development of a New Method for Assessing Global Risk of Alzheimer's Disease for Use in Population Health Approaches to Prevention. Prevention science 2013;14(4):411-21.
25. Schiepers OJG, Kohler S, Deckers K, et al. Lifestyle for Brain Health (LIBRA): a new model for dementia prevention. International journal of geriatric psychiatry. 2018;33(1):167-75.
26. Anstey KJ, Kootar S, Huque MH, et al. Development of the CogDrisk tool to assess risk factors for dementia. Alzheimer's & Dementia: Diagnosis, Assessment & Disease Monitoring. 2022;14(1):e12336.
27. D'Agostino Sr RB, Vasan RS, Pencina MJ, et al. General cardiovascular risk profile for use in primary care: the Framingham Heart Study. Circulation. 2008;117(6):743-53.
28. Pencina MJ, D'Agostino Sr RB, Larson MG, et al. Predicting the 30-year risk of cardiovascular disease: the Framingham Heart Study. Circulation. 2009;119(24):3078-84.
29. Chen L, Magliano DJ, Balkau B, et al. AUSDRISK: an Australian Type 2 Diabetes Risk Assessment Tool based on demographic, lifestyle and simple anthropometric measures. Medical Journal of Australia. 2010;192(4):197-202.
30. Lindstrom J, Tuomilehto J. The diabetes risk score: a practical tool to predict type 2 diabetes risk. Diabetes care. 2003;26(3):725-31.
31. Griffin S, Little P, Hales C, et al. Diabetes risk score: towards earlier detection of type 2 diabetes in general practice. Diabetes/metabolism research and reviews. 2000;16(3):164-71.
32. Wilson PW, Meigs JB, Sullivan L, et al. Prediction of incident diabetes mellitus in middle-aged adults: the Framingham Offspring Study. Archives of internal medicine. 2007;167(10):1068-74.
33. Fried LP, Borhani NO, Enright P, et al. The cardiovascular health study: design and rationale. Annals of epidemiology. 1991;1(3):263-76.
34. Dawber TR, Meadors GF, Moore Jr FE. Epidemiological approaches to heart disease: the Framingham Study. American Journal of Public Health and the Nations Health. 1951;41(3):279-86.
35. Tsao CW, Vasan RS. Cohort Profile: The Framingham Heart Study (FHS): overview of milestones in cardiovascular epidemiology. International journal of epidemiology. 2015;44(6):1800-13.
36. Satizabal CL, Beiser AS, Chouraki V, et al. Incidence of dementia over three decades in the Framingham Heart Study. New England Journal of Medicine. 2016;374(6):523-32.
37. Brayne C, McCracken C, Matthews FE. Cohort profile: the Medical Research Council cognitive function and ageing study (CFAS). International Journal of Epidemiology. 2006;35(5):1140-5.
38. Matthews FE, Stephan BC, Robinson L, et al. A two decade dementia incidence comparison from the Cognitive Function and Ageing Studies I and II. Nature communications. 2016;7(1):11398.
39. CFAS-II. Study design and protocol [Available from: http://www.cfas.ac.uk/cfas-ii/cfasii-study-design/.
40. Sachdev PS, Brodaty H, Reppermund S, et al. The Sydney Memory and Ageing Study (MAS): methodology and baseline medical and neuropsychiatric characteristics of an elderly epidemiological non-demented cohort of Australians aged 70–90 years. International psychogeriatrics. 2010;22(8):1248-64.
41. Jolles J, Houx P, Van Boxtel M, et al. The Maastricht Aging Study. Determinants of cognitive aging Maastricht: Neuropsych Publishers. 1995:192.

For peer review only

For peer review only - http://bmjopen.bmj.com/site/about/guidelines.xhtml

42. Schievink SH, van Boxtel MP, Deckers K, et al. Cognitive changes in prevalent and incident cardiovascular disease: a 12-year follow-up in the Maastricht Aging Study (MAAS). European heart journal. 2022;43(7):e2-e9.
43. Sonnega A, Faul JD, Ofstedal MB, et al. Cohort profile: the health and retirement study (HRS). International journal of epidemiology. 2014;43(2):576-85.
44. Hudomiet P, Hurd MD, Rohwedder S. Dementia prevalence in the United States in 2000 and 2012: Estimates based on a nationally representative study. The Journals of Gerontology: Series B. 2018;73(suppl_1):S10-S9.
45. Plassman BL, Langa KM, Fisher GG, et al. Prevalence of dementia in the United States: the aging, demographics, and memory study. Neuroepidemiology. 2007;29(1-2):125-32.
46. Bennett DA, Schneider JA, Buchman AS, et al. The Rush Memory and Aging Project: study design and baseline characteristics of the study cohort. Neuroepidemiology. 2005;25(4):163-75.
47. ARIC-Investigators. The Atherosclerosis Risk in Communities (ARIC) study: design and objectives. Am J Epidemiol. 1989;129(4):687-702.
48. Knopman DS, Gottesman RF, Sharrett AR, et al. Mild cognitive impairment and dementia prevalence: the atherosclerosis risk in communities neurocognitive study. Alzheimer's & Dementia: Diagnosis, Assessment & Disease Monitoring. 2016;2:1-11.
49. Niti M, Yap K-B, Kua E-H, et al. Physical, social and productive leisure activities, cognitive decline and interaction with APOE-ε4 genotype in Chinese older adults. International psychogeriatrics. 2008;20(2):237-51.
50. Heywood R, Gao Q, Nyunt MSZ, et al. Hearing loss and risk of mild cognitive impairment and dementia: findings from the Singapore longitudinal ageing study. Dementia and geriatric cognitive disorders. 2017;43(5-6):259-68.
51. Matthews FE, Arthur A, Barnes LE, et al. A two-decade comparison of prevalence of dementia in individuals aged 65 years and older from three geographical areas of England: results of the Cognitive Function and Ageing Study I and II. The Lancet. 2013;382(9902):1405-12.
52. Ives DG, Fitzpatrick AL, Bild DE, et al. Surveillance and ascertainment of cardiovascular events: the Cardiovascular Health Study. Annals of epidemiology. 1995;5(4):278-85.
53. Bellou V, Belbasis L, Tzoulaki I, et al. Risk factors for type 2 diabetes mellitus: an exposure-wide umbrella review of meta-analyses. PloS one. 2018;13(3):e0194127.
54. Psaltopoulou T, Sergentanis TN, Panagiotakos DB, et al. Mediterranean diet, stroke, cognitive impairment, and depression: a meta-analysis. Annals of neurology. 2013;74(4):580-91.
55. Wahid A, Manek N, Nichols M, et al. Quantifying the association between physical activity and cardiovascular disease and diabetes: a systematic review and meta-analysis. Journal of the American Heart Association. 2016;5(9):e002495.
56. Huang Y, Cai X, Li Y, et al. Prehypertension and the risk of stroke: a meta-analysis. Neurology. 2014;82(13):1153-61.
57. Lu Y, Hajifathalian K, Ezzati M, et al. Metabolic mediators of the effects of body-mass index, overweight, and obesity on coronary heart disease and stroke: a pooled analysis of 97 prospective cohorts with 1· 8 million participants. Lancet (London, England). 2013;383(9921):970-83.
58. Fine JP, Gray RJ. A proportional hazards model for the subdistribution of a competing risk. Journal of the American statistical association. 1999;94(446):496-509.
59. Austin PC, Lee DS, D'Agostino RB, et al. Developing points-based risk-scoring systems in the presence of competing risks. Statistics in medicine. 2016;35(22):4056-72.
60. Pencina MJ, D'Agostino Sr RB, D'Agostino Jr RB, et al. Evaluating the added predictive ability of a new marker: from area under the ROC curve to reclassification and beyond. Statistics in medicine. 2008;27(2):157-72.

For peer review only - http://bmjopen.bmj.com/site/about/guidelines.xhtml

61. Anstey KJ, Byles JE, Luszcz MA, et al. Cohort profile: the dynamic analyses to optimize ageing (DYNOPTA) project. International Journal of Epidemiology. 2010;39(1):44-51.
62. Van Buuren S, Eyres S, Tennant A, et al. Response conversion: A new technology for comparing existing health information: Leiden: TNO; 2001.
63. WHO. Global recommendations on physical activity for health. Geneva: World Health Organisation; 2010.
64. Lloyd-Jones DM, Hong Y, Labarthe D, et al. Defining and setting national goals for cardiovascular health promotion and disease reduction: the American Heart Association's strategic Impact Goal through 2020 and beyond. Circulation. 2010;121(4):586-613.
65. NHMRC. Australian Guidelines to Reduce Health Risks from Drinking Alcohol. 2020.
66. NHS-UK. High cholesterol 2023 [Available from: https://www.nhsinform.scot/illnesses-and-conditions/blood-and-lymph/high-cholesterol.
67. Expert Panel on Detection E. Executive summary of the third report of the National Cholesterol Education Program (NCEP) expert panel on detection, evaluation, and treatment of high blood cholesterol in adults (Adult Treatment Panel III). Jama. 2001;285(19):2486-97.
68. Huque MH, Carlin JB, Simpson JA, et al. A comparison of multiple imputation methods for missing data in longitudinal studies. BMC medical research methodology. 2018;18(1):1-16.
69. White IR, Royston P, Wood AM. Multiple imputation using chained equations: issues and guidance for practice. Statistics in medicine. 2011;30(4):377-99.
70. Ensor J, Snell KI, Martin EC. PMCALPLOT: Stata module to produce calibration plot of prediction model performance. 2020.

For peer review only

For peer review only - http://bmjopen.bmj.com/site/about/guidelines.xhtml

TRIPOD Checklist: Prediction Model Development and Validation

Section/Topic	Item		Checklist Item	Page
**Title and abstract**				
Title	1	D;V	Identify the study as developing and/or validating a multivariable prediction model, the target population, and the outcome to be predicted.	1
Abstract	2	D;V	Provide a summary of objectives, study design, setting, participants, sample size, predictors, outcome, statistical analysis, results, and conclusions.	2
**Introduction**				
Background and objectives	3a	D;V	Explain the medical context (including whether diagnostic or prognostic) and rationale for developing or validating the multivariable prediction model, including references to existing models.	4
	3b	D;V	Specify the objectives, including whether the study describes the development or validation of the model or both.	6
**Methods**				
Source of data	4a	D;V	Describe the study design or source of data (e.g., randomized trial, cohort, or registry data), separately for the development and validation data sets, if applicable.	6-7
	4b	D;V	Specify the key study dates, including start of accrual; end of accrual; and, if applicable, end of follow-up.	Table 1
Participants	5a	D;V	Specify key elements of the study setting (e.g., primary care, secondary care, general population) including number and location of centres.	7
	5b	D;V	Describe eligibility criteria for participants.	7
	5c	D;V	Give details of treatments received, if relevant.	-
Outcome	6a	D;V	Clearly define the outcome that is predicted by the prediction model, including how and when assessed.	7
	6b	D;V	Report any actions to blind assessment of the outcome to be predicted.	-
Predictors	7a	D;V	Clearly define all predictors used in developing or validating the multivariable prediction model, including how and when they were measured.	9
	7b	D;V	Report any actions to blind assessment of predictors for the outcome and other predictors.	-
Sample size	8	D;V	Explain how the study size was arrived at.	10
Missing data	9	D;V	Describe how missing data were handled (e.g., complete-case analysis, single imputation, multiple imputation) with details of any imputation method.	13
Statistical analysis methods	10a	D	Describe how predictors were handled in the analyses.	12-13
	10b	D	Specify type of model, all model-building procedures (including any predictor selection), and method for internal validation.	11
	10c	V	For validation, describe how the predictions were calculated.	12-13
	10d	D;V	Specify all measures used to assess model performance and, if relevant, to compare multiple models.	14
	10e	V	Describe any model updating (e.g., recalibration) arising from the validation, if done.	14
Risk groups	11	D;V	Provide details on how risk groups were created, if done.	-
Development vs. validation	12	V	For validation, identify any differences from the development data in setting, eligibility criteria, outcome, and predictors.	-
**Results**				
Participants	13a	D;V	Describe the flow of participants through the study, including the number of participants with and without the outcome and, if applicable, a summary of the follow-up time. A diagram may be helpful.	Table 1
	13b	D;V	Describe the characteristics of the participants (basic demographics, clinical features, available predictors), including the number of participants with missing data for predictors and outcome.	-
	13c	V	For validation, show a comparison with the development data of the distribution of important variables (demographics, predictors and outcome).	-
Model development	14a	D	Specify the number of participants and outcome events in each analysis.	-
	14b	D	If done, report the unadjusted association between each candidate predictor and outcome.	-
Model specification	15a	D	Present the full prediction model to allow predictions for individuals (i.e., all regression coefficients, and model intercept or baseline survival at a given time point).	-
	15b	D	Explain how to the use the prediction model.	-
Model performance	16	D;V	Report performance measures (with CIs) for the prediction model.	-
Model-updating	17	V	If done, report the results from any model updating (i.e., model specification, model performance).	-
**Discussion**				
Limitations	18	D;V	Discuss any limitations of the study (such as nonrepresentative sample, few events per predictor, missing data).	16
Interpretation	19a	V	For validation, discuss the results with reference to performance in the development data, and any other validation data.	-
	19b	D;V	Give an overall interpretation of the results, considering objectives, limitations, results from similar studies, and other relevant evidence.	17
Implications	20	D;V	Discuss the potential clinical use of the model and implications for future research.	17
**Other information**				
Supplementary information	21	D;V	Provide information about the availability of supplementary resources, such as study protocol, Web calculator, and data sets.	-
Funding	22	D;V	Give the source of funding and the role of the funders for the present study.	18

*Items relevant only to the development of a prediction model are denoted by D, items relating solely to a validation of a prediction model are denoted by V, and items relating to both are denoted D;V. We recommend using the TRIPOD Checklist in conjunction with the TRIPOD Explanation and Elaboration document.

BMJ Open

Study protocol for development and validation of a single tool to assess risks of stroke, diabetes mellitus, myocardial infarction, and dementia: DemNCD-Risk

Journal:	*BMJ Open*
Manuscript ID	bmjopen-2023-076860.R1
Article Type:	Protocol
Date Submitted by the Author:	03-Sep-2023
Complete List of Authors:	Kootar, Scherazad; Neuroscience Research Australia; University of New South Wales, School of Psychology<br>Huque, Md Hamidul; University of New South Wales, School of Psychology; Neuroscience Research Australia<br>Kiely, Kim; University of New South Wales, School of Psychology; Neuroscience Research Australia<br>Anderson, Craig; George Institute for Global Health, The George Institute for Global Health<br>Jorm, Louisa; University of New South Wales, Centre for Big Data Research in Health<br>Kivipelto, Miia; Karolinska Institutet, Division of Geriatric Epidemiology<br>Lautenschlager, Nicola; University of Melbourne Academic Unit for Psychiatry of Old Age, Academic Unit of Psychiatry of Old Age<br>Matthews, Fiona; Newcastle University, Population Health Sciences Institute<br>Shaw, Jonathan; Baker Heart and Diabetes Institute<br>Whitmer, Rachel; University of California Davis<br>Peters, Ruth; University of New South Wales,<br>Anstey, Kaarin; University of New South Wales, School of Psychology; Neuroscience Research Australia
**Primary Subject Heading**:	Public health
Secondary Subject Heading:	Diabetes and endocrinology, Neurology, Evidence based practice, Epidemiology
Keywords:	Risk Factors, Primary Prevention, Dementia, Stroke < NEUROLOGY, DIABETES & ENDOCRINOLOGY, Myocardial infarction < CARDIOLOGY

SCHOLARONE™
Manuscripts

Study protocol for development and validation of a single tool to assess risks of stroke, diabetes mellitus, myocardial infarction, and dementia: DemNCD-Risk

Scherazad Kootar[1,2,3], Md Hamidul Huque[1,2,3], Kim Kiely[1,2,3], Craig S. Anderson[5], Louisa Jorm[6], Miia Kivipelto[7,8,9,10], Nicola T. Lautenschlager[11,12], Fiona E Matthews[13], Jonathan E. Shaw[14], Rachel A. Whitmer[15], Ruth Peters[2,3,4,5], *Kaarin J. Anstey[1,2,3]

1. Neuroscience Research Australia, Sydney, Australia
2. School of Psychology, University of New South Wales, Sydney, Australia
3. UNSW Ageing Futures Institute, University of New South Wales, Sydney, Australia
4. School of Public Health, Imperial College, London, Exhibition Road, South Kensington, London SW 2AZ UK
5. The George Institute for Global Health, University of New South Wales, Sydney, Australia
6. Centre for Big Data Research in Health, University of New South Wales, Sydney, Australia
7. The Ageing Epidemiology Research Unit, School of Public Health, Imperial College London, London, United Kingdom
8. Division of Clinical Geriatrics, Department of Neurobiology, Center for Alzheimer's Research, Care Sciences and Society, Karolinska Institute, Stockholm, Sweden
9. Theme Inflammation and Aging, Karolinska University Hospital, Stockholm, Sweden
10. Institute of Public Health and Clinical Nutrition, University of Eastern Finland, Kuopio, Finland
11. Academic Unit for Psychiatry of Old Age, Department of Psychiatry, The University of Melbourne, Parkville, Australia
12. North Western Mental Health, Royal Melbourne Hospital, Parkville, Australia
13. Population Health Sciences Institute, Newcastle University, UK
14. Baker Heart and Diabetes Institute, Melbourne, Australia
15. Department of Public Health Sciences and Neurology, University of California, Davis CA 95616, USA

***Corresponding author**:

Scientia Professor Kaarin J. Anstey
School of Psychology,
University of New South Wales,
Kensington NSW 2052, Australia.
Email: k.anstey@unsw.edu.au
Telephone no: +61 9399 1061

Word count: 3,609

For peer review only - http://bmjopen.bmj.com/site/about/guidelines.xhtml

ABSTRACT

Introduction

Current efforts to reduce dementia focus on prevention and risk reduction by targeting modifiable risk factors. As dementia and cardiometabolic non-communicable diseases (NCDs) share risk factors, a single risk-estimating tool for dementia and multiple NCDs could be cost-effective and facilitate concurrent assessments as compared to a conventional single approach. The aim of this study is to develop and validate a new risk tool that estimates an individual's risk of developing dementia and other NCDs including diabetes mellitus, stroke, and myocardial infarction. Once validated, it could be used by the public and General Practitioners.

Methods and Analysis

Ten high-quality cohort studies from multiple countries were identified that met eligibility criteria, including large representative samples, long-term follow-up, data on clinical diagnoses of dementia and NCDs, recognised modifiable risk factors for the four NCDs, and mortality data. Pooled harmonized data from the cohorts will be used, with 65% randomly allocated for development of the predictive model and 35% for testing. Predictors include social-demographic characteristics, general health risk factors, and lifestyle/behavioural risk factors. A sub-distribution hazard model will assess the risk factors' contribution to the outcome, adjusting for competing mortality risks. Point-based scoring algorithms will be built using predictor weights, internally validated, and the discriminative ability and calibration of the model will be assessed for the outcomes. Sensitivity analyses will include recalculating risk scores using logistic regression.

Ethics and dissemination

Ethics approval is provided by the University of New South Wales Human Research Ethics Committee (UNSW HREC; protocol numbers HC200515, HC3413). All data are de-identified and securely stored on servers at Neuroscience Research Australia. Study findings will be presented at conferences and published in peer-reviewed journals. The tool will be accessible as a public health resource. Knowledge translation and implementation work will explore strategies to apply the tool in clinical practice.

Keywords: risk prediction tool, dementia, stroke, diabetes mellitus, myocardial infarction

For peer review only - http://bmjopen.bmj.com/site/about/guidelines.xhtml

Strengths and limitations of this study

- This international and multi-cohort collaboration draws on data from multiple countries including Australia, Netherlands, Singapore, UK, and USA.
- To our knowledge this will be one of the first validated risk-prediction tools for multiple NCDs, including dementia, that accounts for competing risks and draws on datasets from diverse geographic regions.
- External validation will be required to assess generalisability to under-represented communities, low-resource settings, and other populations not included in the contributing studies.
- We rely on post-hoc harmonisation of risk factors, and not all risk factor measures are directly comparable across studies.

For peer review only

For peer review only - http://bmjopen.bmj.com/site/about/guidelines.xhtml

INTRODUCTION

Non communicable diseases (NCDs) are a global health issue leading to around 41 million deaths each year. Amongst these, cardiovascular diseases account for most deaths (17.9 million), followed by cancer (9.3 million), respiratory illness (4.1 million) and diabetes (1.5 million) (1). According to the Centers for Disease Control and Prevention, USA, dementia ranked seventh for mortality in 2020, alongside the above diseases partially due to global population ageing (2). Dementia is the largest contributor to the non-fatal burden of disease amongst older adults in Australia (3). The Global Burden of Diseases, Injuries, and Risk Factors (GBD) Study 2019 (4) reports the estimated global number of people with dementia increasing from 57 million in 2019 to 153 million in 2050. This increase in the number of people with dementia will negatively impact on individuals, families, health care systems, and society. Given that there is no cure or effective treatment to stop the progression of dementia, the field has shifted focus towards prevention and risk reduction to reduce the burden on health and social care systems. Evidence on modifiable risk factors for dementia has been comprehensively synthesized into recent umbrella reviews (5, 6), a Lancet Commission (7, 8) and WHO Guidelines (9). These reviews show that many NCDs are also risk factors for dementia, including diabetes mellitus (DM), stroke, and other cardiovascular diseases (CVD) (5, 6). In addition, these four NCDs also share common social and lifestyle related risk factors (10). Given the overlap in risk between dementia and other NCDs, the development of common risk reduction strategies may be more cost-effective and efficient, than conventionally approaching each disease individually.

For peer review only - http://bmjopen.bmj.com/site/about/guidelines.xhtml

Risk assessments for NCDs are widely used in clinical practice, research, and policymaking (11-14). The Framingham Risk Score, a well-known example of a health risk assessment for cardiovascular disease risk, was adapted to calculate the absolute CVD risk and is used in the clinical practice guidelines and recommendations of many countries to guide treatment for the primary prevention of CVD (15). Risk assessments primarily identify individuals at high risk of developing the disease and determine preventive measures for reducing risk. They are also used to evaluate risk reduction interventions (15, 16), and population modelling of risk levels (17, 18). Currently there are numerous risk tools available for dementia (19), DM (20), heart disease (21) and stroke (22). Examples of scoring systems used to predict dementia include the Cardiovascular Risk Factors, Aging, and Incidence of Dementia (CAIDE), the ANU-Alzheimer's Disease Risk Index (ANU-ADRI), and the Cognitive Health and Dementia Risk Reduction (CogDrisk) (23-26). Similarly, for the cardiometabolic field there are Framingham risk scores for cardiovascular disease (10-year and 30-year risk) (27, 28), the Australian type 2 Diabetes Risk Assessment tool (AUSDRISK) (29), the FINDRISC Diabetes risk tool (30), the Cambridge Diabetes Risk Score (31), the Framingham diabetes risk score to assess risk of diabetes mellitus (29, 32) and the Framingham stroke risk score (22). Variables such as lack of physical activity, unhealthy diet, smoking, hypertension, and obesity are relevant to dementia and overlap with risk factors for diabetes mellitus and cardiovascular diseases. To our knowledge there is no single risk prediction tool for NCDs that includes dementia. Building such a tool that accounts for substantial overlap in risk factors and interactions between NCDs may be

For peer review only - http://bmjopen.bmj.com/site/about/guidelines.xhtml

cost-effective and save clinical assessment time as compared to assessing each disease individually.

To address the need for a population-based prediction tool for incidence of multiple NCDs including dementia, we propose the development and validation of the DemNCD-Risk tool. This risk tool will provide a combined estimate for an individual's risk of developing dementia, type 2 diabetes mellitus, stroke and myocardial infarction. We anticipate that a multi-domain risk tool that accounts for shared and competing risk will be more likely to be used in primary care as it would reduce assessment time. This study protocol outlines the analytical approach used to develop and validate our risk tool.

METHODS AND ANALYSIS

Study design

Prior to commencing the study, we determined the minimum sample size required and types of cohorts that would be suitable. Inclusion criteria were: population-based cohort studies of mid-life and/or older age participants, with at least 5 years follow-up (exception being CFAS-II with follow up time of 2 years), having clinical diagnosis of dementia and other NCDs and the availability of the majority of risk factors used in current prediction tools. We included CFAS-II due to its similarity to CFAS-I in terms of being based in England and having a comparable population and characteristics. Moreover, the availability of accessible data of CFAS-II along with CFAS-I supported our rationale for including this study in our analysis.

For peer review only - http://bmjopen.bmj.com/site/about/guidelines.xhtml

We identified potential datasets through searches of consortia websites, databases, word of mouth, and consultation with experts. We evaluated cohorts in terms of the outcome measures including studies with the availability of clinical diagnosis of dementia and other NCDs, risk factors and measures (which will also be referred to as predictors in the methodology), length of follow-up time, sample size requirements, and the availability of data from the study custodians. We shortlisted ten cohort studies that met our criteria: the Cardiovascular Health Study (CHS) (33), the Framingham Heart Study (FHS) (34-36), the MRC Cognitive Function and Ageing Studies (CFAS-I (37, 38) and CFAS-II (39)), the Sydney Memory and Aging Study (MAS) (40), the Maastricht Aging Study (MAAS) (41, 42), the Health and Retirement Study- Aging, Demographics and Memory Study (HRS ADAMS) (43-45), the Memory and Aging Project (MAP) (46), the Atherosclerosis Risk in Communities (ARIC) (47, 48) and the Singapore Longitudinal Ageing Studies-I (SLAS I) (49, 50). Key characteristics for each study are available in Table 1. Ethics approval was obtained from the UNSW Human Research Ethics Committee (approval number HC00515) and approval from the data custodians were obtained to access the datasets.

We report the study protocol following the Transparent Reporting of a multivariable prediction model for Individual Prognosis or Diagnosis (TRIPOD) statement for prediction studies.

Patient and Public Involvement

Patients and public were not involved in the design, conduct, reporting or dissemination plans of our research.

For peer review only - http://bmjopen.bmj.com/site/about/guidelines.xhtml

Participants

Individual data will be pooled from the ten cohort studies (age 44 to 110 years). Participants from these cohorts who have dementia at baseline will be excluded.

Outcomes

The primary objective of the current study is to develop a validated risk assessment tool to predict four different NCDs including dementia, myocardial infarction (MI), stroke and DM. We chose to predict for MI instead of coronary heart disease (CHD) as only CHS and FHS had a composite variable for CHD which was adjudicated by a subcommittee while MI was reported in all studies. These NCDs were chosen as they are highly prevalent, related, and they share common modifiable risk factors that are often assessed and managed in primary care. Clinical diagnosis of each of these outcomes will be used (where available), however self-reports of diagnosis may also be used for stroke, diabetes, and MI. Detailed descriptions of each of these outcomes are presented below and described in Table 2:

Dementia: All included studies diagnosed dementia through the Diagnostic and Statistical Manual of Mental Disorders (DSM-III-R, IV criteria) or International Classification of Diseases (ICD) codes. We chose to study all- type dementia instead of specific types for two reasons: one, as there are no tools available to understand the different dementia pathologies to predict dementia types; and two, as we intend to build a tool, which is simple and facilitates public participation. Algorithms were used for diagnosis of dementia in ARIC (48) and CFAS (51).

Stroke: For the ARIC study, a diagnostic algorithm created variable that classifies stroke events based on data from reports will be used to define stroke. For the CHS, special

For peer review only - http://bmjopen.bmj.com/site/about/guidelines.xhtml

cerebrovascular subcommittees defined the criteria for stroke and TIA (52) while in the FHS, stroke cases were reviewed by neurologists. Where clinical diagnosis is not available, for example CFAS I and II, Sydney MAS, SLAS-I and MAAS, and HRS ADAMS self-reported stroke will be used.

Diabetes mellitus: Similar to stroke, clinical diagnosis of DM (including type of diabetes where information is available), derived variables or lab results will be preferred over self-reported DM. Derived variables for diabetes based on WHO guidelines or American Diabetes Association guidelines are available in the CHS while self-reported type 2 diabetes will be considered for other studies.

MI: All the studies report self-reported heart attacks.

Death: Date/year of death or age at death are available in all studies.

Predictors

We identified model predictors in three stages. Firstly, we identify predictors from the latest seminal systematic reviews for the four outcomes (5, 6, 10, 53-57), the Lancet Commission on dementia (7, 8), and the WHO Guidelines for evidence on risk factors and risk reduction of cognitive decline and dementia (9). Secondly, predictors from commonly used risk tools were collated into a long list. For this, we referred to the ANU-ADRI, LIBRA, and CAIDE for dementia predictors, the Framingham hard coronary heart disease risk tool (10-year risk for myocardial infarction or coronary death), Framingham risk score for cardiovascular disease (10-year and 30- year), and CVD check for myocardial infarction predictors, the AUSDRISK and Framingham diabetes risk score for diabetes predictors, and the Framingham stroke risk score for stroke predictors

For peer review only - http://bmjopen.bmj.com/site/about/guidelines.xhtml

(refer Table 3). Lastly, the long list of potential predictors was then reviewed and voted by eight subject matter experts who had previous experience with population-based prediction models (24, 29). Ethics approval was obtained from UNSW Human Research Ethics Committee (protocol number HC3413). We received vote from six experts for dementia predictors, three each for predictors of stroke, diabetes and myocardial infarction. We selected all of the predictors that were nominated by any two of the reviewers and were available in the dataset. In total, we identified 18 predictors for stroke, 15 for diabetes, 18 for coronary heart disease and 23 for dementia. Predictors were mainly extracted from the baseline examination. In cases where this was not available, predictors were selected from the first wave in which they were measured.

Candidate predictors considered for inclusion in the predictive model (definitions are provided in Table 4):

- *Demographic factors*: age, sex, education level, ethnicity/country of birth, socioeconomic status, and family history.
- *Lifestyle risk/protective factors*: smoking, harmful alcohol consumption, fish intake/healthy diet, insufficient physical activity, social isolation, lack of cognitive engagement and sleep disturbance.
- *Medical risk factors*: mid-life obesity, hypertension, hypercholesterolemia, triglycerides, low and high density lipoproteins, late-life atrial fibrillation, chronic kidney disease, high blood glucose, urinary albumin and protein, left ventricular hypertrophy, depression, traumatic brain injury, high homocysteine, stress, orthostatic hypotension, and hearing loss.

Table 4 reports the risk factors and their definitions.

For peer review only - http://bmjopen.bmj.com/site/about/guidelines.xhtml

Sample size

We assumed that the proposed risk algorithms that generate risk scores for dementia and other outcomes will have sensitivity between 70% and 90% across different cohorts as the prevalence of disease will vary across cohorts. Assuming global prevalence of dementia at age 60 and older is 5%, we estimated that 9 studies with sample sizes between 1,041 and 2,165 will be needed to obtain a sensitivity of 70% and 90% respectively with 10% precision and 5% level of significance with Bonferroni correction. We focused on dementia for sample size calculations, as among all the outcomes considered for this study, dementia has the lowest prevalence. Therefore, the minimum sample size for dementia will be sufficient to provide required statistical power for other outcomes.

Statistical analysis methods

<u>Analysis plan</u>

We will pool harmonized individual data from the ten studies to develop a risk tool that provides long term risk estimates for diabetes, stroke, MI, and dementia. Machine learning methods will be used to develop and validate the risk tools. Specifically, we will use 65% of the pooled data in training the model, and the remaining 35% of the data will serve as test data for internal validation in order to ensure good coverage of the outcomes in the validation sample (58). We plan to validate the tool on additional datasets in future work. As our objective is to build a tool that predicts long term health outcomes, we will use competing risk survival models (Fine and Gray sub-distribution hazard model (59)). When predicting outcomes over long periods of time, competing

For peer review only - http://bmjopen.bmj.com/site/about/guidelines.xhtml

risks should account for risk of death from non-disease related causes as this risk increases over time and decedents are no longer at risk of a disease incidence. As the competing risk of mortality is more common in high-risk populations with a long duration of follow-up, this effect may be more pronounced in older cohorts than in younger or healthy cohorts. Moreover, this model allows one to estimate the effect of covariates on the cumulative incidence for the event of interest. In addition, such model needs to incorporate time-varying covariates or to include the factor of time. Since we are using different longitudinal cohorts, not all the time-varying covariates may be available in the longitudinal follow-up in every cohort. In this case, we will develop risk prediction models targeting mid-life (aged 45 to 65 years) and late-life (65 years onward). Predictor weights from this model for risk factor exposure and their interactions will be combined to build point-based scoring algorithms that provide individualized risk prediction (60). The individual risk scores will be validated internally using the area under the curve (AUC) (61). Model calibration will also be carried out. We will estimate sensitivity and specificity of each risk algorithm based on a suitable cut-off on the ROC curve.

Data harmonisation and coding of predictors

Each study wave will be screened for the availability of risk factors and outcomes using the data dictionaries. Predictors and outcomes listed above will be checked for availability of the measure in each wave of the different datasets. Data harmonisation will be carried out as described previously (62) using the 'by fiat' method (63). This method is developed using a common scoring system across studies to standardize the measure of the variables. In certain cases, for example cognitive engagement, we will

For peer review only - http://bmjopen.bmj.com/site/about/guidelines.xhtml

capture the total cognitive activity per study as compared to harmonising the variable across the studies as different items of cognitive activity are measured in each study. Detailed definitions and measurements of the predictors are presented in Table 4.

We will use national / international guidelines where possible, for example; 150 minutes of moderate and vigorous physical activity per week which is equivalent to >500 MET minutes per week of physical activity (64), 5 or more servings of vegetables or 3 -5 servings of fruit and vegetables and more than 2 servings of fish per week will be used to represent a low risk diet (as measured by the AHA's Life's Simple 7 guidelines (65), alcohol consumption of not more than 10 standard drinks (1 standard drink = 10 grams of pure alcohol) per week as recommended by the Australian guidelines (66). For biological markers, we will use cut-offs informed by national and international clinical practice guidelines, and recommendations from national healthcare providers for example the Royal Australia College of General Practitioners (13, 67, 68). Cut-offs to be used: high-density lipoprotein (<1 mmol/L for men and <1.3 mmol/L for women), low density lipoprotein (>4.1 mmol/L), high cholesterol (>6.18mmol/L) and triglycerides (>2.3mmol/L). We will choose "high" instead of "borderline high" as the most robust category for the prediction of disease outcomes. Self-report stress measures will be used, clinical thresholds for high homocysteine, urinary albumin and protein if reported in articles from the cohort studies to be included.

<u>Missing data</u>

Missing data are inevitable in studies with long follow-up and may lead to bias. Variables with missing data will be imputed through multiple imputation across all studies (69). Specifically, a multivariate normal imputation (MVNI) or imputation by fully

For peer review only - http://bmjopen.bmj.com/site/about/guidelines.xhtml

conditional specification, where appropriate, will be employed to impute missing data. Compatibility/congeniality between imputation model and analysis model will be ensured. The number of imputations will be determined according to the current guidelines (70).

<u>Model specification</u>

Following multiple imputation, competing risk regression with available covariates (candidate covariates that are available in the dataset) and death as a competing event will be modelled for each outcome, stratified by sex. Robust sandwich estimation of the variance will be obtained. All the cohorts under consideration are likely to be heterogeneous in terms of sample sizes and underlying population characteristics i.e., outcome and covariate distribution. Moreover, running competing risk analysis with multiple imputed datasets on large number of data may cause the slow convergence of the program. Therefore, we will run the competing risk regression model in each of the datasets separately. As we plan to evaluate the competing risk model in individual datasets, we will not be able to create 10-year risk prediction estimates.

<u>Model estimation</u>

Estimates of the regression models for multiple imputed models will be combined using Rubin's rule in each cohort. The resulting regression estimates will be combined across cohorts to get final estimates using meta-analysis . The regression coefficients estimates from meta-analysis will then be used to calculate the points-based risk score for males and females separately (26), which we will then test with the testing datasets

For peer review only - http://bmjopen.bmj.com/site/about/guidelines.xhtml

for prediction of each of the outcomes (60). All the analyses will be carried out using Stata Version 16.0.

Assessment of Predictive performance

Area under the curve (AUC), sensitivity and specificity of the risk scores on the test dataset will be obtained to assess the discriminatory power. We will also assess the calibration of the risk algorithms using calibration plots and Hosmer-Lemeshow statistics (71).

Model presentation

The final regression coefficients (95% CI) for dementia, stroke, diabetes and MI, along with the points corresponding to the final algorithm will be made available.

Analysis beyond initial model development

In addition to the complex survival model, we will also calculate risk scores from the cohorts under consideration using logistic regression to test for the effect of follow-up time on risk scores. Specifically, we will be modelling binary outcomes with all the predictors ignoring follow-up time for the initial model. The risk score will then be calculated using the regression coefficients from the logistic model using a similar methodology.

In addition to the above model with all the risk factors, we are also planning to build a model based on the best subset of predictors. Specifically, for each of outcome, we are planning to perform backward elimination ($p<0.1$). The risk scores for each outcome will then be calculated using the regression coefficients obtained from the most

For peer review only

For peer review only - http://bmjopen.bmj.com/site/about/guidelines.xhtml

parsimonious model. The AUC (95% CI) of the most parsimonious model will then be compared with the AUC obtained from the full model.

Translation of the analyses into a risk assessment for implementation

To generate information on risk of dementia, DM, stroke and MI using a single assessment, we will create a single set of questions that encompass all the risk factors so that the patient experience is a single assessment. Algorithms will be applied to this tool to generate advice and risk estimates for multiple outcomes. Ideally the analyses of the cohort studies will provide a risk algorithm for each outcome. However, this may be supplemented by existing risk algorithms if the results of the proposed analyses are not sufficient. Where possible, risk factors will be assessed as measured in the original cohort studies. Where the quality of measures is mixed or of suboptimal quality, we will identify validated measures. For example, with respect to the measurement of insomnia, some studies use clinical sleep scales and others include a study-specific self-report item. We will use a standardized and validated instrument for diagnosis of insomnia in the assessment tool. We anticipate that two assessment tools will be created: one for the clinicians and another for researchers and the public. This is due to the fact that clinicians have limited consultation time; their questionnaire will be shorter as compared to the public one.

LIMITATIONS

Our study has some limitations. Although using multiple cohorts to develop the tool is an improvement compared with a single cohort, our cohorts are drawn mainly from high-

For peer review only - http://bmjopen.bmj.com/site/about/guidelines.xhtml

income countries and with most of them being mainly Caucasian due to the availability of these datasets with the required information. This will limit the generalizability of the tool. We are unable to take account of the differences in treatment and care that will have influenced participants in different cohorts.

However, as the tool will be developed and validated from data pooled from America, Asia, Australia and Europe, we have some diversity within our sample. As the tool will be validated on the same sample as the derivation cohort, external validation of the tool will be desirable after study completion. We are unable to account for cause of death in the analysis as most of the studies lack this information.

Moreover, as we will be using secondary data from various cohort studies to estimate the risk of dementia, stroke, MI and diabetes, therefore, the specific types of diagnosis of above conditions may not be available in all studies (e.g., ischemic vs haemorrhagic stroke, type-I vs type-II diabetes etc.). In addition, the method of diagnosis may vary across different studies. This may cause potential biases. To assess the impact of this and other cohort specific biases, we will study the cohort specific prediction in addition to aggregate predictions for all cohorts.

ETHICS AND MODEL DISSEMNINATION

Ethics approval is provided by the University of New South Wales Human Research Ethics Committee (UNSW HREC; protocol numbers HC200515, HC3413). All data are de-identified and stored on a secure server at Neuroscience Research Australia. Study findings will be presented at relevant national and international conferences and

For peer review only - http://bmjopen.bmj.com/site/about/guidelines.xhtml

published in peer review journals. The tool will be made available as a public health resource. Knowledge translation and implementation work will explore strategies to apply the tool in clinical practice.

For peer review only

For peer review only - http://bmjopen.bmj.com/site/about/guidelines.xhtml

Authors' contributions

KJA is the lead investigator of this study and oversaw the project development, co-developed the original project idea; contributed towards developing the analysis plan, study design including identifying various cohort studies, applied to various studies and got approval, and planned the harmonisation of outcomes and predictors. Critically reviewed and contributed to the protocol and approved the final draft.

RP co-developed the original project idea; contributed towards developing the analysis plan, study design including identifying various cohort studies and planned the harmonisation of outcomes and predictors. Critically reviewed and contributed to the protocol and approved the final draft.

SK contributed towards developing the analysis plan, study design including identifying various cohort studies, applied to various studies and got approval, and planned the harmonisation of outcomes and predictors. Reviewed the literature, conducted the survey on subject matter experts for selecting predictors and prepared the first draft of the manuscript. Critically reviewed and contributed to the protocol and approved the final draft.

HH contributed towards developing the analysis plan, study design including identifying various cohort studies, planning the harmonisation of outcomes and predictors and drafted the method section. Critically reviewed and contributed to the protocol and approved the final draft.

For peer review only - http://bmjopen.bmj.com/site/about/guidelines.xhtml

KK contributed towards developing the analysis plan, study design including identifying various cohort studies, and planning the harmonisation of outcomes and predictors. Critically reviewed and contributed to the protocol and approved the final draft.

CSA provided inputs to the analysis plan, critically reviewed and contributed to the protocol and approved the final draft.

LJ provided inputs to the analysis plan, critically reviewed and contributed to the protocol and approved the final draft.

NTL provided inputs to the analysis plan, critically reviewed and contributed to the protocol and approved the final draft.

FEM provided inputs to the analysis plan, critically reviewed and contributed to the protocol and approved the final draft.

JES provided inputs to the analysis plan, critically reviewed and contributed to the protocol and approved the final draft.

RAW provided inputs to the analysis plan, critically reviewed and contributed to the protocol and approved the final draft.

MK provided inputs to the analysis plan, critically reviewed and contributed to the protocol and approved the final draft.

Funding

This research was funded by a NeuRA Grant to KJA, the NHMRC Dementia Collaborative Research Centre, and NHMRC GNT1171279. KJA is funded by the Australian Research Council Fellowship FL190100011, SK and HH are part funded by

For peer review only - http://bmjopen.bmj.com/site/about/guidelines.xhtml

NHMRC GNT1171279. KK is part funded by ARC CE170100005. JES is supported by NHMRC Investigator Grant APP1173952. CSA holds research grants and a Senior Investigator Fellowship from the NHMRC.

Competing interests

JES has received honoraria for scientific advisory, lectures, and clinical research from Pfizer; Roche; Zuellig Pharma; Astra Zeneca; Sanofi; Novo Nordisk; MSD; Eli Lilly; Abbott; Mylan; Boehringer Ingelheim. CSA has received grants from Takeda, Penumbra and Credit pharma awarded to his institution outside of this work.

For peer review only

For peer review only - http://bmjopen.bmj.com/site/about/guidelines.xhtml

Table 1: Characteristics of contributing studies

**Study**	**Baseline year**	**Age range at baseline**	**Number of individuals at baseline**	**Males (n) and Females (n)**	**Number of waves and year**	**Follow up time**	**Country**
Maastricht Aging Study (MAAS)	1992	24 - 81 years	1,823	Women= 910, Men=913	Baseline (1992-1996), Wave 1 (1996-1999), Wave 2 (1999-2002), Wave 3 (2002-2005), Wave 4 (2005-2008) Wave 5 (2018-2020)	25 years	The Netherlands
The Framingham Heart Study (FHS)	1948	30 -62 years	5,209	Women =2,873, Men =2,336	Wave 1 (1948-1953) to Wave 32 (2012-2014)	61 years	USA
Atherosclerosis Risk in Communities (ARIC)	1985	44-66 years	15,028	Women =8,198, Men =6,830	Wave 1 (1987-1989), Wave 2 (1990-1992), Wave 3 (1993-1995), Wave 4 (1996-1998), Wave 5 (2011-2013), Wave 6 (2015-2016), Wave 7 (2017-2018), Wave 8 (2019-2020)	31 years	USA
Health and Retirement Study - Aging, Demographics and Memory Study (HRS ADAMS)	1992	70 years and above	856	Women =501, Men=355	Wave 1 (2002-2005), Wave 2 (2006-2008), Wave 3 (2008-2009)	8 years	USA
The Singapore Longitudinal Ageing Study (SLAS I)	2003	55 years and above	2,567	Women =1,666, Men= 901	Wave 1 (2003-2005) and Wave 2 (2007-2009)	6 years	Singapore

For peer review only

The Cardiovascular Health Study (CHS)	1991-1994	65-100 years	5,888	Women =3,393, Men =2,495	Wave 1 (1991-1994) Final follow-up (1999-2000)	8 years	USA
Cognitive Function and Ageing Study (CFAS-I)	1989-1991	65 -105 years and above	13,004	Women =7,847, Men = 5,157	Wave 1 (1991-1994), Wave 2 (1991-1995), Wave 3 (1992-1996), Wave 4 (1994-1998), Wave 5 (1997-2001)	10 years	UK
Cognitive Function and Ageing Study (CFAS-II)	2008	65 -100 years and above	7,762	Women =4,228, Men =3,534	Wave 1 (2008-2011) Wave 2 (2011-2013)	2 years	UK
Sydney Memory and Aging Study (MAS)	2005	70-90 years	1,037	Women = 572, Men =465	Wave 1 (2005-2007), Wave 2 (2007-2009), Wave 3 (2009-2011), Wave 4 (2011-2013), Wave 5 (2013-2016), Wave 6 (2016-2018), Wave 7 (2018 onwards)	12 years	Australia
Rush Memory and Aging Project (MAP)	1997	55 years and above	2,192	Women =1,610, Men =582	Wave 1 (1998) to Wave 22 (2019 and onwards)	22 years	USA

For peer review only - http://bmjopen.bmj.com/site/about/guidelines.xhtml

Table 2: Considerations for defining the outcomes

No	Outcome	Considerations
1	Dementia	Clinical diagnosis of dementia
2	Stroke	Clinical diagnosis of stroke, if not available then self-reported stroke
3	Diabetes	Clinical diagnosis of diabetes or derived variable or lab results, if not available then use self-reported Diabetes.
4	MI	Clinical diagnosis of myocardial infarction, if not available use self-reported questions

For peer review only

Table 3: List of predictors included in current risk tools for dementia, diabetes, myocardial infarction and stroke

For peer review only - http://bmjopen.bmj.com/site/about/guidelines.xhtml

Risk factor category	Dementia risk tools (CAIDE, ANU ADRI, LIBRA)	Cardiovascular risk tools (Framingham Risk Tools- Hard Coronary Heart Disease, 10- /30-year risk of CVD, CVD Check)	Diabetes risk tools (AUSDRISK, Framingham risk score for Diabetes)	Stroke risk tool (Framingham stroke risk score)
Demographic variables				
Age and Sex	Y	Y	Y	Y
Education	Y	-	-	-
Ethnicity	-	Y	Y	-
Parental /family history of disease	-	Y	Y	-
Medical risk factors				
Hypertension/ Systolic BP	Y	Y	Y	Y
Use of antihypertensives	-	Y	Y	Y
High total cholesterol	Y	Y	-	-
HDL cholesterol	-	Y	Y	-
Obesity/ Waist circumference	Y	Y	Y	-
Diabetes	Y	Y	-	Y
Depression	Y	-	-	-
Traumatic Brain Injury	Y	-	-	-
High blood glucose levels	-	-	Y	-
Triglycerides	-	-	Y	-
Chronic kidney disease/renal dysfunction	Y	Y	-	-
Urine for microalbumin and protein	-	Y	-	-
Atrial fibrillation	-	Y	-	Y
Prior cardiovascular disease	Y	-	-	Y
Left ventricular hypertrophy	-	-	-	Y

For peer review only - http://bmjopen.bmj.com/site/about/guidelines.xhtml

Lifestyle and behavioural risk factors				
Smoking	Y	Y	Y	Y
Physical inactivity	Y	Y	Y	-
Diet (Fish intake, Consumption of vegetables and fruits)	Y	Y	Y	-
Alcohol consumption	Y	Y	-	-
Social engagement	Y	-	-	-
Cognitive engagement	Y	-	-	-
Environmental exposure				
Pesticide exposure	Y	-	-	-

For peer review only

For peer review only - http://bmjopen.bmj.com/site/about/guidelines.xhtml

Table 4: Considerations for defining the candidate predictors

Predictors	Types of predictors	Considerations
Demographic factors		
Education	Categorical	Less than secondary vs Secondary and vocational training vs Tertiary
Ethnicity and country of birth	Categorical	Questions related to race/ethnicity and country of birth
Socioeconomic status	Categorical	Questions on occupation, income and accommodation
Family history	Binary	Family History of stroke, diabetes, dementia, MI
Lifestyle risk factors		
Smoking status	Binary	Current vs Former vs Never
Alcohol consumption	Categorical	Abstinence vs drinker<br>Average number of drinks per drinking session<br>Heavy drinker vs non heavy drinker
Fish intake / Healthy diet	Binary	Fish intake- more than twice per week<br>Fruit and vegetable intake (at least 5 servings of vegetables or 3 servings of vegetables and 2 servings of fruits)
Physical inactivity	Binary	Total time per week of physical activity- more than 500 MET per week -physically active vs inactive<br>Sedentary- yes vs no
Social isolation	Binary	Loneliness vs not lonely
Cognitive engagement	Categorical	Decided to use the Total cognitive activity as reported by each study and then to divided into tertiles/quartiles- depending on each study to get a cut-off for cognitive inactivity.
Sleep disturbance	Binary	Account for those with at least two symptoms of the three self-reported sleep problems: trouble falling asleep, wake up several times at night, wake up too early
Medical risk factors		
BMI categories	Categorical	WHO classifications- Underweight (BMI <18.5) vs Normal (BMI 18.5-24.9) vs Overweight (BMI 25-29.9) vs Obese (BMI >30)
Hypertension status	Binary	Yes -Blood pressure average systolic >=140mm Hg OR average diastolic >=90 OR history of hypertension OR currently using antihypertensives vs No
High Cholesterol and triglycerides	Binary	High cholesterol (self-reported/lifetime history or total cholesterol >6.18 mmol/l)
		High triglycerides (>2.3 mmol/L triglycerides)
		High cholesterol or high triglycerides (self-reported)
Low density lipoproteins	Binary	> 4.1 mmol/L of LDL cut-off is high LDL

For peer review only - http://bmjopen.bmj.com/site/about/guidelines.xhtml

High density lipoprotein	Binary	< 1 mmol/L (for males) and <1.3 mmol/L (for females) of HDL cut-off is low HDL
Atrial fibrillation	Binary	AF measured by ECG or self-reported history of AF
Chronic kidney disease	Binary	First preference Clinical diagnosis of kidney disease, if not use self-reported history of kidney disease
High blood glucose	Binary	To consider borderline or prediabetes cut off for blood glucose - 100 to 125 mg/dl or 5.6-7.0 mmol/L
Urinary microalbumin and protein	Categorical	Lab reports of urine microalbumin and protein levels
Left Ventricular Hypertrophy	Binary	Yes or no for LVH as defined by study
Depression	Binary	Self-reported depression symptoms measured by respective scales of depression used by the respective studies
Traumatic brain injury	Binary	Self-reported history of brain injury/head injury/knock out with or without loss of consciousness
High homocysteine	Binary	Lab reports of homocysteine levels
Stress	Binary	Self-reported stress measures
Orthostatic hypotension	Binary	Use self-reported orthostatic hypotension or calculated variable
Hearing loss	Binary	To categorise in three groups - no hearing loss, has hearing loss and wears hearing aid, has hearing loss but does not wear a hearing aid.

Abbreviations:
MI: Myocardial infarction
MET: Metabolic Equivalent of Task
WHO: World Health Organisation
BMI: Body Mass Index
LDL: Low Density Lipoprotein
HDL: High Density Lipoprotein
AF: Atrial Fibrillation
LVH: Left Ventricular Hypertrophy

For peer review only

For peer review only - http://bmjopen.bmj.com/site/about/guidelines.xhtml

References:

1. WHO. Noncommunicable diseases 2021 [Available from: https://www.who.int/news-room/fact-sheets/detail/noncommunicable-diseases.
2. Murphy SL, Kochanek KD, Xu J, et al. Mortality in the United States, 2020. 2021.
3. AIHW. Australian Burden of Disease Study: impact and causes of illness and death in Australia 2018. Cat no BOD 29. 2021.
4. Nichols E, Szoeke CE, Vollset SE, et al. Global, regional, and national burden of Alzheimer's disease and other dementias, 1990–2016: a systematic analysis for the Global Burden of Disease Study 2016. The Lancet Neurology. 2019;18(1):88-106.
5. Anstey KJ, Ee N, Eramudugolla R, et al. A Systematic Review of Meta-Analyses that Evaluate Risk Factors for Dementia to Evaluate the Quantity, Quality, and Global Representativeness of Evidence. Journal of Alzheimer's disease : JAD. 2019;70(s1):S165-s86;10.3233/jad-190181.
6. Yu J-T, Xu W, Tan C-C, et al. Evidence-based prevention of Alzheimer's disease: systematic review and meta-analysis of 243 observational prospective studies and 153 randomised controlled trials. Journal of Neurology, Neurosurgery & Psychiatry. 2020;91(11):1201-9.
7. Livingston G, Huntley J, Sommerlad A, et al. Dementia prevention, intervention, and care: 2020 report of the Lancet Commission. The Lancet. 2020;396(10248):413-46.
8. Livingston G, Sommerlad A, Orgeta V, et al. Dementia prevention, intervention, and care. The Lancet. 2017;390(10113):2673-734.
9. WHO. Risk Reduction of Cognitive Decline and Dementia: WHO guidelines Geneva: World Health Organisation; 2019.
10. Peters R, Booth A, Rockwood K, et al. Combining modifiable risk factors and risk of dementia: a systematic review and meta-analysis. BMJ Open. 2019;9(1):e022846;10.1136/bmjopen-2018-022846.
11. Duerden M, O'Flynn N, Qureshi N. Cardiovascular risk assessment and lipid modification: NICE guideline. British Journal of General Practice. 2015;65(636):378-80.
12. Kaptoge S, Pennells L, De Bacquer D, et al. World Health Organization cardiovascular disease risk charts: revised models to estimate risk in 21 global regions. The Lancet Global Health. 2019;7(10):e1332-e45.
13. Litt J. RACGP Guidelines for preventive activities in general practice 9th edition. 2016.
14. Visvanathan K, Hurley P, Bantug E, et al. Use of pharmacologic interventions for breast cancer risk reduction: American Society of Clinical Oncology clinical practice guideline. Journal of Clinical Oncology. 2013;31(23):2942-62.
15. NVDPA. Guidelines for the management of absolute cardiovascular disease risk. 2012.
16. Anstey KJ, Cherbuin N, Kim S, et al. An internet-based intervention augmented with a diet and physical activity consultation to decrease the risk of dementia in at-risk adults in a primary care setting: pragmatic randomized controlled trial. Journal of medical Internet research. 2020;22(9):e19431.
17. Ng R, Sutradhar R, Kornas K, et al. Development and validation of the Chronic Disease Population Risk Tool (CDPoRT) to predict incidence of adult chronic disease. JAMA network open. 2020;3(6):e204669-e.
18. Hajifathalian K, Ueda P, Lu Y, et al. A novel risk score to predict cardiovascular disease risk in national populations (Globorisk): a pooled analysis of prospective cohorts and health examination surveys. The lancet Diabetes & endocrinology. 2015;3(5):339-55.
19. Hou X-H, Feng L, Zhang C, et al. Models for predicting risk of dementia: a systematic review. Journal of Neurology, Neurosurgery & Psychiatry. 2019;90(4):373-9.
20. Noble D, Mathur R, Dent T, et al. Risk models and scores for type 2 diabetes: systematic review. Bmj. 2011;343.

For peer review only - http://bmjopen.bmj.com/site/about/guidelines.xhtml

21. Damen JA, Hooft L, Schuit E, et al. Prediction models for cardiovascular disease risk in the general population: systematic review. bmj. 2016;353.
22. D'Agostino RB, Wolf PA, Belanger AJ, et al. Stroke risk profile: adjustment for antihypertensive medication. The Framingham Study. Stroke. 1994;25(1):40-3.
23. Kivipelto M, Ngandu T, Laatikainen T, et al. Risk score for the prediction of dementia risk in 20 years among middle aged people: a longitudinal, population-based study. The Lancet Neurology. 2006;5(9):735-41.
24. Anstey KJ, Cherbuin N, Herath PM. Development of a New Method for Assessing Global Risk of Alzheimer's Disease for Use in Population Health Approaches to Prevention. Prevention science 2013;14(4):411-21;10.1007/s11121-012-0313-2.
25. Schiepers OJG, Kohler S, Deckers K, et al. Lifestyle for Brain Health (LIBRA): a new model for dementia prevention. International journal of geriatric psychiatry. 2018;33(1):167-75;10.1002/gps.4700.
26. Anstey KJ, Kootar S, Huque MH, et al. Development of the CogDrisk tool to assess risk factors for dementia. Alzheimer's & Dementia: Diagnosis, Assessment & Disease Monitoring. 2022;14(1):e12336.
27. D'Agostino Sr RB, Vasan RS, Pencina MJ, et al. General cardiovascular risk profile for use in primary care: the Framingham Heart Study. Circulation. 2008;117(6):743-53.
28. Pencina MJ, D'Agostino Sr RB, Larson MG, et al. Predicting the 30-year risk of cardiovascular disease: the Framingham Heart Study. Circulation. 2009;119(24):3078-84.
29. Chen L, Magliano DJ, Balkau B, et al. AUSDRISK: an Australian Type 2 Diabetes Risk Assessment Tool based on demographic, lifestyle and simple anthropometric measures. Medical Journal of Australia. 2010;192(4):197-202.
30. Lindstrom J, Tuomilehto J. The diabetes risk score: a practical tool to predict type 2 diabetes risk. Diabetes care. 2003;26(3):725-31.
31. Griffin S, Little P, Hales C, et al. Diabetes risk score: towards earlier detection of type 2 diabetes in general practice. Diabetes/metabolism research and reviews. 2000;16(3):164-71.
32. Wilson PW, Meigs JB, Sullivan L, et al. Prediction of incident diabetes mellitus in middle-aged adults: the Framingham Offspring Study. Archives of internal medicine. 2007;167(10):1068-74.
33. Fried LP, Borhani NO, Enright P, et al. The cardiovascular health study: design and rationale. Annals of epidemiology. 1991;1(3):263-76.
34. Dawber TR, Meadors GF, Moore Jr FE. Epidemiological approaches to heart disease: the Framingham Study. American Journal of Public Health and the Nations Health. 1951;41(3):279-86.
35. Tsao CW, Vasan RS. Cohort Profile: The Framingham Heart Study (FHS): overview of milestones in cardiovascular epidemiology. International journal of epidemiology. 2015;44(6):1800-13.
36. Satizabal CL, Beiser AS, Chouraki V, et al. Incidence of dementia over three decades in the Framingham Heart Study. New England Journal of Medicine. 2016;374(6):523-32.
37. Brayne C, McCracken C, Matthews FE. Cohort profile: the Medical Research Council cognitive function and ageing study (CFAS). International Journal of Epidemiology. 2006;35(5):1140-5.
38. Matthews FE, Stephan BC, Robinson L, et al. A two decade dementia incidence comparison from the Cognitive Function and Ageing Studies I and II. Nature communications. 2016;7(1):11398.
39. CFAS-II. Study design and protocol [Available from: http://www.cfas.ac.uk/cfas-ii/cfasii-study-design/.
40. Sachdev PS, Brodaty H, Reppermund S, et al. The Sydney Memory and Ageing Study (MAS): methodology and baseline medical and neuropsychiatric characteristics of an elderly epidemiological non-demented cohort of Australians aged 70–90 years. International psychogeriatrics. 2010;22(8):1248-64.
41. Jolles J, Houx P, Van Boxtel M, et al. The Maastricht Aging Study. Determinants of cognitive aging Maastricht: Neuropsych Publishers. 1995:192.

For peer review only - http://bmjopen.bmj.com/site/about/guidelines.xhtml

42. Schievink SH, van Boxtel MP, Deckers K, et al. Cognitive changes in prevalent and incident cardiovascular disease: a 12-year follow-up in the Maastricht Aging Study (MAAS). European heart journal. 2022;43(7):e2-e9.
43. Sonnega A, Faul JD, Ofstedal MB, et al. Cohort profile: the health and retirement study (HRS). International journal of epidemiology. 2014;43(2):576-85.
44. Hudomiet P, Hurd MD, Rohwedder S. Dementia prevalence in the United States in 2000 and 2012: Estimates based on a nationally representative study. The Journals of Gerontology: Series B. 2018;73(suppl_1):S10-S9.
45. Plassman BL, Langa KM, Fisher GG, et al. Prevalence of dementia in the United States: the aging, demographics, and memory study. Neuroepidemiology. 2007;29(1-2):125-32.
46. Bennett DA, Schneider JA, Buchman AS, et al. The Rush Memory and Aging Project: study design and baseline characteristics of the study cohort. Neuroepidemiology. 2005;25(4):163-75.
47. ARIC-Investigators. The Atherosclerosis Risk in Communities (ARIC) study: design and objectives. Am J Epidemiol. 1989;129(4):687-702.
48. Knopman DS, Gottesman RF, Sharrett AR, et al. Mild cognitive impairment and dementia prevalence: the atherosclerosis risk in communities neurocognitive study. Alzheimer's & Dementia: Diagnosis, Assessment & Disease Monitoring. 2016;2:1-11.
49. Niti M, Yap K-B, Kua E-H, et al. Physical, social and productive leisure activities, cognitive decline and interaction with APOE-ε4 genotype in Chinese older adults. International psychogeriatrics. 2008;20(2):237-51.
50. Heywood R, Gao Q, Nyunt MSZ, et al. Hearing loss and risk of mild cognitive impairment and dementia: findings from the Singapore longitudinal ageing study. Dementia and geriatric cognitive disorders. 2017;43(5-6):259-68.
51. Matthews FE, Arthur A, Barnes LE, et al. A two-decade comparison of prevalence of dementia in individuals aged 65 years and older from three geographical areas of England: results of the Cognitive Function and Ageing Study I and II. The Lancet. 2013;382(9902):1405-12.
52. Ives DG, Fitzpatrick AL, Bild DE, et al. Surveillance and ascertainment of cardiovascular events: the Cardiovascular Health Study. Annals of epidemiology. 1995;5(4):278-85.
53. Bellou V, Belbasis L, Tzoulaki I, et al. Risk factors for type 2 diabetes mellitus: an exposure-wide umbrella review of meta-analyses. PloS one. 2018;13(3):e0194127.
54. Psaltopoulou T, Sergentanis TN, Panagiotakos DB, et al. Mediterranean diet, stroke, cognitive impairment, and depression: a meta-analysis. Annals of neurology. 2013;74(4):580-91.
55. Wahid A, Manek N, Nichols M, et al. Quantifying the association between physical activity and cardiovascular disease and diabetes: a systematic review and meta-analysis. Journal of the American Heart Association. 2016;5(9):e002495.
56. Huang Y, Cai X, Li Y, et al. Prehypertension and the risk of stroke: a meta-analysis. Neurology. 2014;82(13):1153-61.
57. Lu Y, Hajifathalian K, Ezzati M, et al. Metabolic mediators of the effects of body-mass index, overweight, and obesity on coronary heart disease and stroke: a pooled analysis of 97 prospective cohorts with 1· 8 million participants. Lancet (London, England). 2013;383(9921):970-83.
58. Hastie T, Tibshirani R, Friedman JH, et al. The elements of statistical learning: data mining, inference, and prediction: Springer; 2009.
59. Fine JP, Gray RJ. A proportional hazards model for the subdistribution of a competing risk. Journal of the American statistical association. 1999;94(446):496-509.
60. Austin PC, Lee DS, D'Agostino RB, et al. Developing points-based risk-scoring systems in the presence of competing risks. Statistics in medicine. 2016;35(22):4056-72.

For peer review only - http://bmjopen.bmj.com/site/about/guidelines.xhtml

61. Pencina MJ, D'Agostino Sr RB, D'Agostino Jr RB, et al. Evaluating the added predictive ability of a new marker: from area under the ROC curve to reclassification and beyond. Statistics in medicine. 2008;27(2):157-72.
62. Anstey KJ, Byles JE, Luszcz MA, et al. Cohort profile: the dynamic analyses to optimize ageing (DYNOPTA) project. International Journal of Epidemiology. 2010;39(1):44-51.
63. Van Buuren S, Eyres S, Tennant A, et al. Response conversion: A new technology for comparing existing health information: Leiden: TNO; 2001.
64. WHO. Global recommendations on physical activity for health. Geneva: World Health Organisation; 2010.
65. Lloyd-Jones DM, Hong Y, Labarthe D, et al. Defining and setting national goals for cardiovascular health promotion and disease reduction: the American Heart Association's strategic Impact Goal through 2020 and beyond. Circulation. 2010;121(4):586-613.
66. NHMRC. Australian Guidelines to Reduce Health Risks from Drinking Alcohol. 2020.
67. NHS-UK. High cholesterol 2023 [Available from: https://www.nhsinform.scot/illnesses-and-conditions/blood-and-lymph/high-cholesterol.
68. Expert Panel on Detection E. Executive summary of the third report of the National Cholesterol Education Program (NCEP) expert panel on detection, evaluation, and treatment of high blood cholesterol in adults (Adult Treatment Panel III). Jama. 2001;285(19):2486-97.
69. Huque MH, Carlin JB, Simpson JA, et al. A comparison of multiple imputation methods for missing data in longitudinal studies. BMC medical research methodology. 2018;18(1):1-16.
70. White IR, Royston P, Wood AM. Multiple imputation using chained equations: issues and guidance for practice. Statistics in medicine. 2011;30(4):377-99.
71. Ensor J, Snell KI, Martin EC. PMCALPLOT: Stata module to produce calibration plot of prediction model performance. 2020.

For peer review only

For peer review only - http://bmjopen.bmj.com/site/about/guidelines.xhtml

TRIPOD Checklist: Prediction Model Development and Validation

Section/Topic	Item		Checklist Item	Page
**Title and abstract**				
Title	1	D;V	Identify the study as developing and/or validating a multivariable prediction model, the target population, and the outcome to be predicted.	1
Abstract	2	D;V	Provide a summary of objectives, study design, setting, participants, sample size, predictors, outcome, statistical analysis, results, and conclusions.	2
**Introduction**				
Background and objectives	3a	D;V	Explain the medical context (including whether diagnostic or prognostic) and rationale for developing or validating the multivariable prediction model, including references to existing models.	4
	3b	D;V	Specify the objectives, including whether the study describes the development or validation of the model or both.	6
**Methods**				
Source of data	4a	D;V	Describe the study design or source of data (e.g., randomized trial, cohort, or registry data), separately for the development and validation data sets, if applicable.	6-7
	4b	D;V	Specify the key study dates, including start of accrual; end of accrual; and, if applicable, end of follow-up.	Table 1
Participants	5a	D;V	Specify key elements of the study setting (e.g., primary care, secondary care, general population) including number and location of centres.	7
	5b	D;V	Describe eligibility criteria for participants.	7
	5c	D;V	Give details of treatments received, if relevant.	-
Outcome	6a	D;V	Clearly define the outcome that is predicted by the prediction model, including how and when assessed.	7
	6b	D;V	Report any actions to blind assessment of the outcome to be predicted.	-
Predictors	7a	D;V	Clearly define all predictors used in developing or validating the multivariable prediction model, including how and when they were measured.	9
	7b	D;V	Report any actions to blind assessment of predictors for the outcome and other predictors.	-
Sample size	8	D;V	Explain how the study size was arrived at.	10
Missing data	9	D;V	Describe how missing data were handled (e.g., complete-case analysis, single imputation, multiple imputation) with details of any imputation method.	13
Statistical analysis methods	10a	D	Describe how predictors were handled in the analyses.	12-13
	10b	D	Specify type of model, all model-building procedures (including any predictor selection), and method for internal validation.	11
	10c	V	For validation, describe how the predictions were calculated.	12-13
	10d	D;V	Specify all measures used to assess model performance and, if relevant, to compare multiple models.	14
	10e	V	Describe any model updating (e.g., recalibration) arising from the validation, if done.	14
Risk groups	11	D;V	Provide details on how risk groups were created, if done.	-
Development vs. validation	12	V	For validation, identify any differences from the development data in setting, eligibility criteria, outcome, and predictors.	-
**Results**				
Participants	13a	D;V	Describe the flow of participants through the study, including the number of participants with and without the outcome and, if applicable, a summary of the follow-up time. A diagram may be helpful.	Table 1
	13b	D;V	Describe the characteristics of the participants (basic demographics, clinical features, available predictors), including the number of participants with missing data for predictors and outcome.	-
	13c	V	For validation, show a comparison with the development data of the distribution of important variables (demographics, predictors and outcome).	-
Model development	14a	D	Specify the number of participants and outcome events in each analysis.	-
	14b	D	If done, report the unadjusted association between each candidate predictor and outcome.	-
Model specification	15a	D	Present the full prediction model to allow predictions for individuals (i.e., all regression coefficients, and model intercept or baseline survival at a given time point).	-
	15b	D	Explain how to the use the prediction model.	-
Model performance	16	D;V	Report performance measures (with CIs) for the prediction model.	-
Model-updating	17	V	If done, report the results from any model updating (i.e., model specification, model performance).	-
**Discussion**				
Limitations	18	D;V	Discuss any limitations of the study (such as nonrepresentative sample, few events per predictor, missing data).	16
Interpretation	19a	V	For validation, discuss the results with reference to performance in the development data, and any other validation data.	-
	19b	D;V	Give an overall interpretation of the results, considering objectives, limitations, results from similar studies, and other relevant evidence.	17
Implications	20	D;V	Discuss the potential clinical use of the model and implications for future research.	17
**Other information**				
Supplementary information	21	D;V	Provide information about the availability of supplementary resources, such as study protocol, Web calculator, and data sets.	-
Funding	22	D;V	Give the source of funding and the role of the funders for the present study.	18

*Items relevant only to the development of a prediction model are denoted by D, items relating solely to a validation of a prediction model are denoted by V, and items relating to both are denoted D;V. We recommend using the TRIPOD Checklist in conjunction with the TRIPOD Explanation and Elaboration document.

For peer review only - http://bmjopen.bmj.com/site/about/guidelines.xhtml
